# Multiomics Identification and Validation of an Integrin–Extracellular Matrix Network Driving Respiratory Syncytial Virus‐Induced Lung Injury and Repair

**DOI:** 10.1002/mco2.70811

**Published:** 2026-07-01

**Authors:** Lili Zhou, Hua Guo, Xiaofeng Yu, Yu Ran, Ruiqi Liu, Jiali Zhu, Ran Li, Jie Xu, Leshi Chen, Yongliang Zhu, Long Zhang, Zhenjiang Bai, Fangfang Zhou

**Affiliations:** ^1^ Center of Clinical Laboratory the First Affiliated Hospital of Soochow University Suzhou China; ^2^ The Institutes of Biology and Medical Science Suzhou Medical College Soochow University Suzhou China; ^3^ School of Medicine Hangzhou City University Hangzhou China; ^4^ State Key Laboratory of Genetic Engineering School of Life Sciences Fudan University Shanghai China; ^5^ Laboratory of Gastroenterology Department Second Affiliated Hospital of Zhejiang University School of Medicine Hangzhou China; ^6^ Department of Radiation Oncology and the State Key Laboratory of Transvascular Implantation Devices, The Second Affiliated Hospital of Zhejiang University School of Medicine, Life Sciences Institute Zhejiang University Hangzhou China; ^7^ Pediatric Intensive Care Unit Children's Hospital of Soochow University Suzhou China; ^8^ Biomedical Basic Research Center (BBRC) of Jiangsu Province Suzhou China

**Keywords:** respiratory syncytial virus (RSV), viral pneumonia, multiomics, integrin signaling, extracellular matrix (ECM)

## Abstract

Respiratory syncytial virus (RSV) is a major cause of severe lung injury, particularly in infants. Most previous studies have relied on single‐omics or single‐model systems, limiting a comprehensive understanding of the dynamic and coordinated host response. To overcome this, we employed an integrated multiomics approach across complementary in vivo (murine) and physiologically relevant ex vivo (human bronchial organoid) models, combining longitudinal transcriptomic and proteomic profiling to systematically delineate the spatiotemporal dynamics of RSV‐induced lung injury and repair. Our analysis revealed a stage‐specific progression from early inflammatory injury to late repair, in which extracellular matrix (ECM)–receptor interaction and PI3K–Akt signaling play central roles. Moreover, a core set of hub genes including *Itgb3*, *Itga2b*, and *Fn1* were positively correlated with RSV‐induced lung injury and clinical disease severity. Pharmacological inhibition of αIIbβ3 (encoded by *Itga2b* and *Itgb3*) or fibronectin (*Fn1*) in vivo significantly attenuated immunopathology and lung injury without affecting viral clearance, directly establishing their causal role in disease pathogenesis. Collectively, our study not only provides novel insights into the integrin‐centric network driving RSV immunopathology but also identifies a potential biomarker panel for clinical severity stratification and therapeutic targets for intervention.

## Graphical abstract

This graphical abstract depicts the dynamic progression from acute lung inflammation to fibrotic repair following RSV infection. Multiomics analysis uncovers a central regulatory network coordinating key pathways including coagulation, extracellular matrix deposition, integrin signaling, and cytoskeletal reorganization.

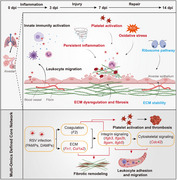

## 1 Introduction

Respiratory syncytial virus (RSV) is a single‐stranded negative‐sense RNA virus belonging to the paramyxoviridae family [1, 2, 3]. It encodes 11 proteins, including two nonstructural proteins (NS1 and NS2), three envelope glycoproteins (fusion F, attachment G, and small hydrophobic SH), one matrix protein (M), four nucleocapsid proteins (N, P, L, M2‐1), and the RNA‐regulatory factor M2‐2 [4, 5, 6]. RSV transmission occurs via respiratory droplets or fomites, infecting the upper respiratory tract through nasopharyngeal or conjunctival mucosa [7]. RSV infection is most prevalent in children under 2 years of age, with severe symptoms observed in premature infants, young children, immunocompromised adults, and the elderly [8, 9, 10]. In these vulnerable populations, mild symptoms mimicking the common cold often progresses to lower respiratory tract infections, pneumonia, bronchitis, and life‐threatening complications, imposing a major burden on healthcare systems worldwide [11, 12, 13].

Despite decades of research, effective antiviral therapies and licensed vaccines remain scarce. Current management is predominantly supportive. Palivizumab, the only approved prophylactic agent, is limited to high‐risk infants [14]. The complex pathophysiology of RSV, particularly the dynamic transition from acute lung injury to repair, is incompletely understood, hindering the development of targeted interventions. Following infection, RSV activates innate immune pathways, triggering the production of reactive oxygen species (ROS), proinflammatory cytokines (e.g., IL‐6, TNF‐α), and chemokines, which recruit neutrophils and macrophages to the lungs [15, 16, 17]. While critical for viral clearance, this response can become dysregulated, evolving into a “cytokine storm” that causes epithelial damage, vascular leakage, alveolar edema, and impaired gas exchange [16, 18]. The host must maintain a delicate balance between viral elimination and avoidance of immunopathology; disruption of this balance exacerbates tissue injury and, in severe cases (especially with bacterial coinfection), can progress to pediatric acute respiratory distress syndrome [17, 19].

Although significant progress has been made in identifying individual molecular pathways and effector cells involved in RSV pathogenesis [20, 21], most previous studies have relied on single‐omics or single‐model systems, which limits a comprehensive understanding of the dynamic and coordinated host response spanning from injury to repair. Integrated multiomics approaches enable systematic delineation of networks from gene expression to protein function, revealing cross‐level interactions that shape disease phenotypes [22, 23]. However, the spatiotemporal dynamics of host molecular programs driving the transition from acute lung injury to repair remain largely uncharacterized in RSV infection. Furthermore, whether specific molecular hubs serve as causal drivers of immunopathology has not been functionally validated.

In this study, we integrated transcriptomic and proteomic analyses using murine in vivo models and human bronchial organoid ex vivo systems to systematically profile dynamic changes in RSV‐triggered lung injury and repair. We identified 7 days postinfection (dpi) as a key time point for the transition from tissue damage to regeneration, alongside marked changes in extracellular matrix (ECM)‐receptor interaction and PI3K–Akt signaling pathways. Protein–protein interaction (PPI) network analysis revealed core hub genes including *Itgb3*, *Itga2b*, and *Fn1* closely associated with RSV‐related lung injury. In vivo pharmacological inhibition of these molecules markedly mitigated pulmonary immunopathology and tissue damage without interfering with viral clearance, thereby verifying their essential pathogenic roles. Additionally, the expression of these integrin–ECM related genes positively correlated with disease severity in RSV‐infected patients and exhibited reliable predictive capacity in machine learning models. Collectively, our results elucidate an integrin‐dominated regulatory network underlying RSV‐induced lung damage, confirm promising therapeutic targets, and provide candidate biomarkers for clinical severity evaluation.

## 2 Results

### 2.1 Integrated Multiomics Uncovers Spatiotemporal Dynamics of Lung Injury

**FIGURE 1:**
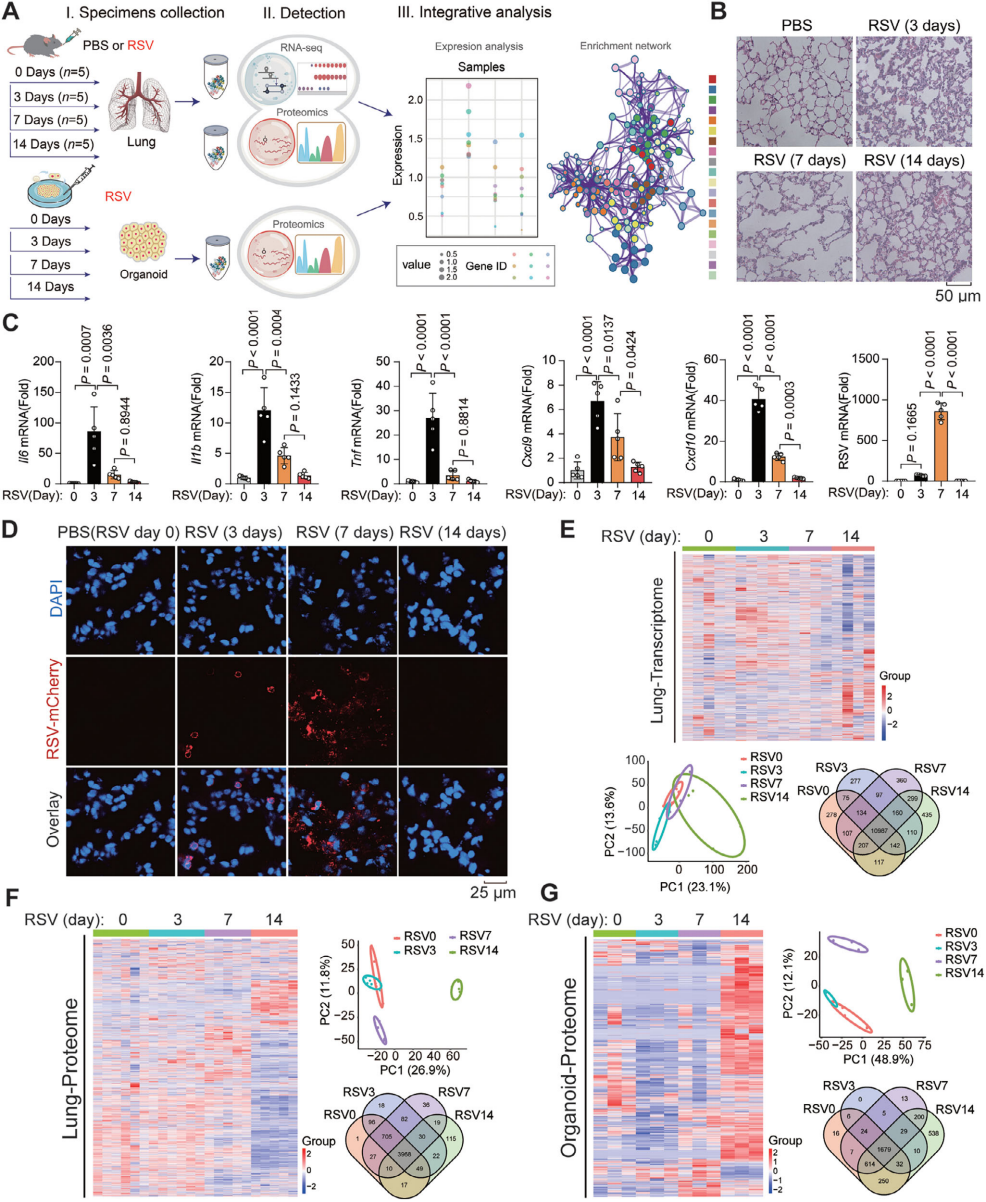
Multiomics landscape of RSV infection samples. (A) Flow chart summarizing the experimental procedures and data analysis workflow. (B) Hematoxylin and eosin (H&E) staining of lung sections from mice infected with RSV–mcherry (1 × 10^8^ PFU) for 0, 3, 7, and 14 days. Scale bars, 50 µm. (C) qPCR analysis of *Il6*, *Il1b*, *Tnf*, *Cxcl9*, *Cxcl10* mRNA, and RSV RNA in RSV‐infected mouse lungs as in (A). *n* = 5 per group. Statistical analysis was performed using two‐way ANOVA. (D) Fluorescence microscopy of RSV–mCherry in RSV‐infected mouse lungs as in (A). Scale bars: 25 µm. (E) Heatmap, PCA, and Venn analyses of differentially expressed features in the transcriptome of RSV‐infected mouse lungs. (F) Heatmap, PCA, and Venn analyses of differentially expressed features in the proteome of RSV‐infected mouse lungs. (G) Heatmap, (PCA, and Venn analyses of differentially expressed features in the proteome of RSV‐infected human lung bronchus.

To characterize the landscape of immune response and lung injury upon RSV infection, we infected C57BL/6 mice with RSV–mCherry (a recombinant RSV strain expressing mCherry fluorescent protein) for 0, 3, 7, and 14 days and performed systematic transcriptomic and proteomic analyses (Figure 1A). Histopathological staining revealed that lung tissues from the control group exhibited no inflammatory cell infiltration, with clear and intact bronchial and alveolar structures. By 3 dpi, significant inflammatory cell infiltration was observed, accompanied by congestion, edema, and marked thickening of the alveolar walls. The extent of lung tissue injury further intensified by 7 dpi, manifested as alveolar wall rupture, fusion, and enlargement of the airspaces. Although mild inflammation persisted at 14 dpi, the lung tissue damage showed significant improvement compared with earlier stages (Figure 1B).

We next quantified the pulmonary expression of key proinflammatory mediators. Real‐Time quantitative PCR (qPCR) analysis demonstrated a robust and transient induction of cytokine and chemokine mRNA levels in infected lungs. Transcripts of *Il6*, *Il1b*, *Tnf*, *Cxcl9*, and *Cxcl10* exhibited a rapid increase, reaching peak expression levels at 3 dpi and a progressive decline, returning to near baseline by 14 dpi (Figure 1C).

RSV replication was increased within the lungs, peaking at 7 dpi, followed by a significant decline, by 14 dpi (Figure 1C). Consistent with this, visualization of RSV–mCherry fluorescence intensity in lung sections confirmed maximal viral burden at 7 dpi, with almost invisible signal by 14 dpi (Figure 1D).

Based on these findings, the four time points of 0, 3, 7, and 14 dpi effectively capture the core progression of RSV infection: from initial immune activation (3 dpi, peak inflammatory cytokine levels), through the peak of viral replication and maximal tissue injury (7 dpi), to viral clearance and the initiation of tissue repair (14 dpi).

A total of 16,989 transcripts were quantified across the mouse lung tissue samples. Heatmap visualization demonstrated high intragroup consistency and clear intergroup heterogeneity among the transcriptomic profiles at different RSV infection time points. Principal component analysis (PCA) further confirmed this, revealing distinct clustering of transcriptomic profiles corresponding to each infection stage. Venn diagram analysis identified 10,987 coexpressed transcripts present across multiple infection time points in these mouse lungs (Figure 1E). In parallel, 5195 proteins were quantified in the same mouse lung samples, with robust intragroup reproducibility and significant intergroup differences. PCA analysis of the proteomes also resulted in clear separation of the samples by infection time point, reinforcing the stage‐specific molecular signatures. For these mouse lung proteins, 3968 common proteins were shared across time points (Figure 1F). To address potential differences in physiological structure and biological characteristics between murine and human lung tissues, parallel infections were performed on a human bronchial organoid model, and proteomic analysis was also conducted. Analysis of the human bronchial organoid model quantified 3423 proteins. The proteomic profiles similarly exhibited high intragroup consistency and marked intergroup heterogeneity across the infection time course, with PCA confirming distinct stage‐specific clustering. In the human bronchial organoids, 1679 common proteins were consistently detected across the infection time course (Figure 1G). These analyses collectively demonstrate high intragroup reproducibility and reveal significant intergroup heterogeneity corresponding to distinct stages of RSV infection, suggesting the reliability of the transcriptomic and proteomic datasets for subsequent analysis of the dynamic host response to infection in both murine and human model systems.

### 2.2 Dynamic Transcriptome Analysis of Lung Injury Caused by RSV

To gain deeper insights into the transcriptional changes in mouse lung tissue induced by RSV infection, we performed differential transcript analysis comparing infected groups at various time points to the control group.

**FIGURE 2:**
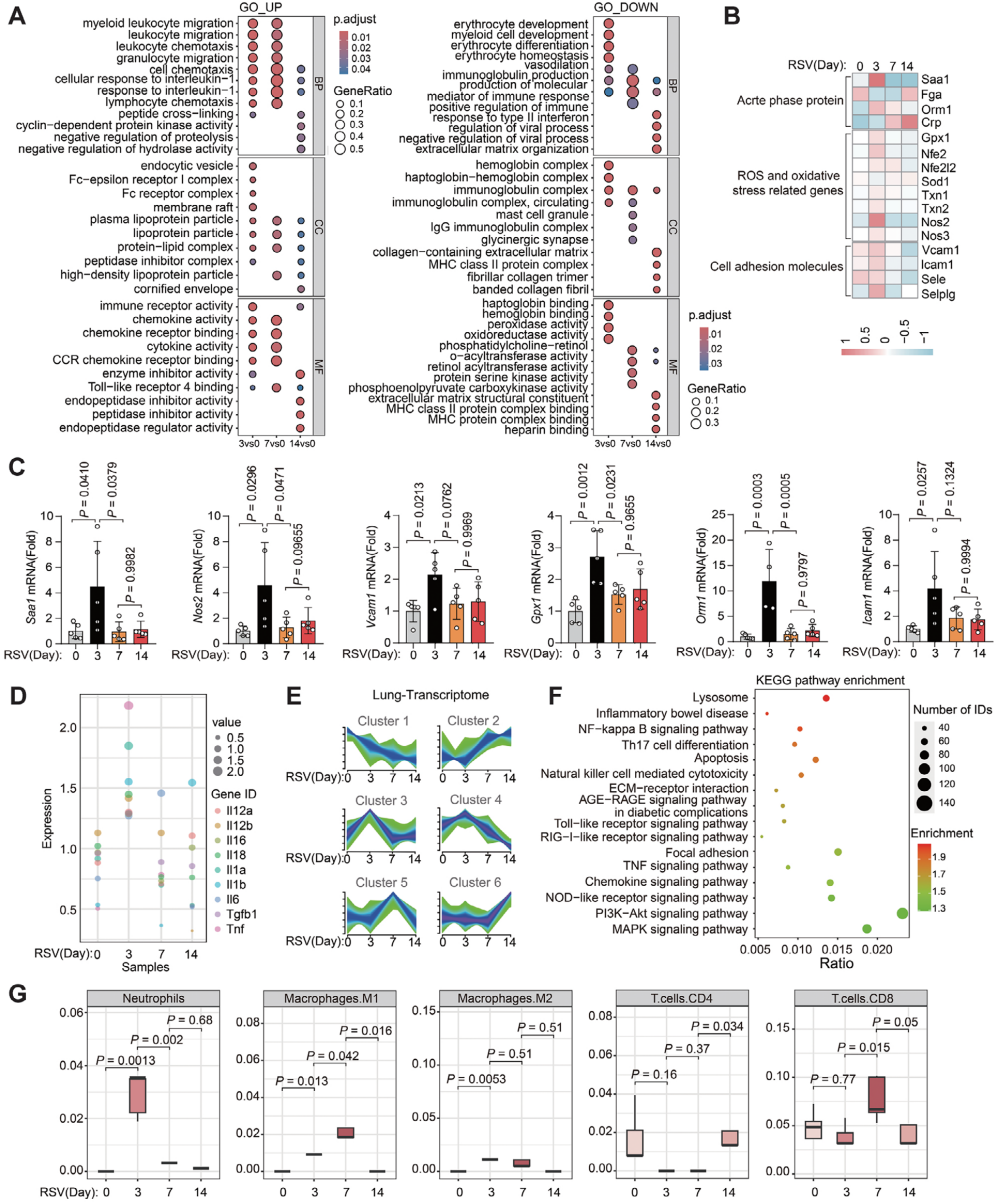
Transcriptomic features in lungs of RSV‐infected mice. (A) Gene Ontology (GO) enrichment analysis of upregulated and downregulated genes in lung tissues at 3, 7, or 14 dpi versus 0 dpi (3vs0, 7vs0, and 14vs0). Bubble charts show enriched terms for biological process (BP), cellular component (CC), and molecular function (MF) categories. (B) Heatmap showing expression levels of lung injury‐associated genes at 0, 3, 7, and 14 dpi. Relative expression is indicated by the color gradient (blue: downregulated; red: upregulated). (C) qPCR validation of lung injury‐associated gene (*Saa1*, *Nos2*, *Vcam1*, *Gpx1*, *Orm1*, *Icam1*) expression identified as in (B). *n* = 5 per group. Statistical analysis was performed using two‐way ANOVA. (D) Transcripts of inflammatory cytokine genes in lung tissues at 0, 3, 7, and 14 dpi. (E) Fuzzy c‐means clustering analysis of transcriptional changes, revealing six distinct expression clusters over the infection time course. Data points represent quantitative mRNA expression from individual mice at each time point. (F) Kyoto Encyclopedia of Genes and Genomes (KEGG) pathway enrichment analysis for gene clusters 3 and 4, associated with disease pathogenesis. Bubble size represents the proportion of target genes in the pathway; color intensity indicates enrichment significance (red: high; green: low). (G) Deconvolution of bulk RNA‐seq data for major immune cell clusters across all samples. Statistical comparisons between groups (*n* = 5 per group) were performed using a two‐sided Wilcoxon test.

At 3 dpi, compared with controls, we identified 127 differentially expressed transcripts (DETs), comprising 98 upregulated and 29 downregulated genes. Upregulated genes were dominantly enriched in proinflammatory pathways, including chemokine activity, leukocyte chemotaxis, cellular response to interleukin‐1, and immune receptor activity. Downregulated genes were enriched in biological processes related to erythrocyte development/differentiation and immunoglobulin production, indicating early suppression of adaptive immunity components. This is consistent with previous reports indicating that RSV infection impairs antibody‐mediated protection and causes IgA B‐cell memory deficiency in the host [23], suggesting that RSV actively interferes with antibody production and immune memory formation (Figure 2A).

At 7 dpi, compared with controls, 74 DETs were found (19 upregulated, 55 downregulated). Upregulated genes continued to show enrichment in inflammatory processes, like chemokine receptor binding and cytokine activity, indicating sustained innate immune activation. Downregulated genes were strongly enriched in immunoglobulin production, protein kinase activity regulation, and positive regulation of immune response. This pronounced downregulation of adaptive immunity genes suggests RSV may induce immune suppression or dysregulation at this stage, alongside persistent inflammation (Figure 2A).

At 14 dpi, compared with controls, 167 DETs were identified (56 upregulated, 111 downregulated). Analysis of upregulated genes revealed two distinct protective mechanisms. First, a strategy centered on tissue stabilization and inflammation suppression: enhanced expression of genes involved in peptide cross‐linking indicates increased extracellular matrix stabilization, while concurrent downregulation of genes associated with proteolysis and hydrolase activity suppresses tissue degradation. Second, signaling for cell cycle reinitiation to promote repair, evidenced by upregulated cyclin‐dependent kinase activity. Downregulated genes were predominantly enriched in type II interferon (IFN‐γ) signaling and MHC class II molecule binding. The concomitant downregulation of both proteolysis‐related genes and IFN‐γ signaling pathways likely functions to mitigate excessive immune responses and limit tissue damage (Figure 2A).

To further elucidate the mechanisms underlying RSV‐induced lung injury, we specifically analyzed the expression of established injury markers. The results demonstrated that the transcriptional levels of these injury markers peaked at 3 dpi (Figure 2B,C), suggesting that significant lung injury occurs early in the infection. Concurrently, the transcriptional levels of proinflammatory cytokines also reached their maximum at 3 dpi (Figure 2D), closely aligning with the acute immune and inflammatory responses. These findings indicate a strong temporal association between the peak expression of lung injury markers and proinflammatory cytokines. Based on these findings, we propose that RSV‐induced lung injury is closely associated with inflammation, potentially involving excessive immune cell activation and dysregulated cytokine secretion.

To explore the genes and signaling pathways implicated in RSV‐induced lung injury progression, we employed the Mfuzz package to analyze the dynamic transcriptional changes across time points. Using fuzzy c‐means clustering, all identified transcripts were partitioned into six distinct dynamic expression clusters (Figures 2E and S1). Strikingly, the expression patterns of Cluster 3 and Cluster 4 exhibited a high degree of concordance with the trajectory of lung injury severity. Pathway enrichment analysis of these clusters revealed significant enrichment of multiple immune‐ and inflammation‐related signaling pathways, including NF‐κB, Toll‐like receptor (TLR), TNF receptor, and RIG‐I signaling pathways (Figure 2F). Furthermore, pathways related to ECM remodeling, PI3K–AKT signaling, and MAPK signaling were also significantly enriched, suggesting that RSV infection may disrupt the balance between lung tissue repair and damage by modulating ECM dynamics, cell proliferation, and cell survival processes.

To gain deeper insights into the cellular drivers of the observed transcriptional changes, we performed immune deconvolution analysis on the RNA‐seq data. This approach quantified the dynamic shifts in immune cell populations within the lung throughout RSV infection (Figure 2G).

The analysis unveiled a highly orchestrated, timed immune response. At 3 dpi, we observed a massive influx of neutrophils, which coincided with the peak of proinflammatory cytokine expression. This was accompanied by a moderate increase in macrophages. By the peak of viral load and tissue damage at 7 dpi, macrophages had become the predominant immune cell type, suggesting their crucial role in both immunopathology and viral clearance at this stage.

Interestingly, the dynamics of T lymphocytes exhibited a biphasic response. Their overall abundance was initially suppressed in the early phase of infection but rebounded thereafter. CD8^+^ T cells reached their maximum infiltration at 7 dpi, likely attempting to control the peak viral replication. In contrast, CD4^+^ T cells peaked later, at 14 dpi, aligning with the resolution of inflammation and the initiation of tissue repair processes. This precise temporal mapping of immune infiltration provides a critical cellular context for the transcriptomic changes observed and underscores the stage‐specific roles of distinct immune subsets in RSV‐induced pathogenesis and recovery.

### 2.3 Dynamic Changes in the Lung Proteome During RSV Infection

Proteomic analysis revealed distinct changes in the lung proteome following RSV infection.

**FIGURE 3:**
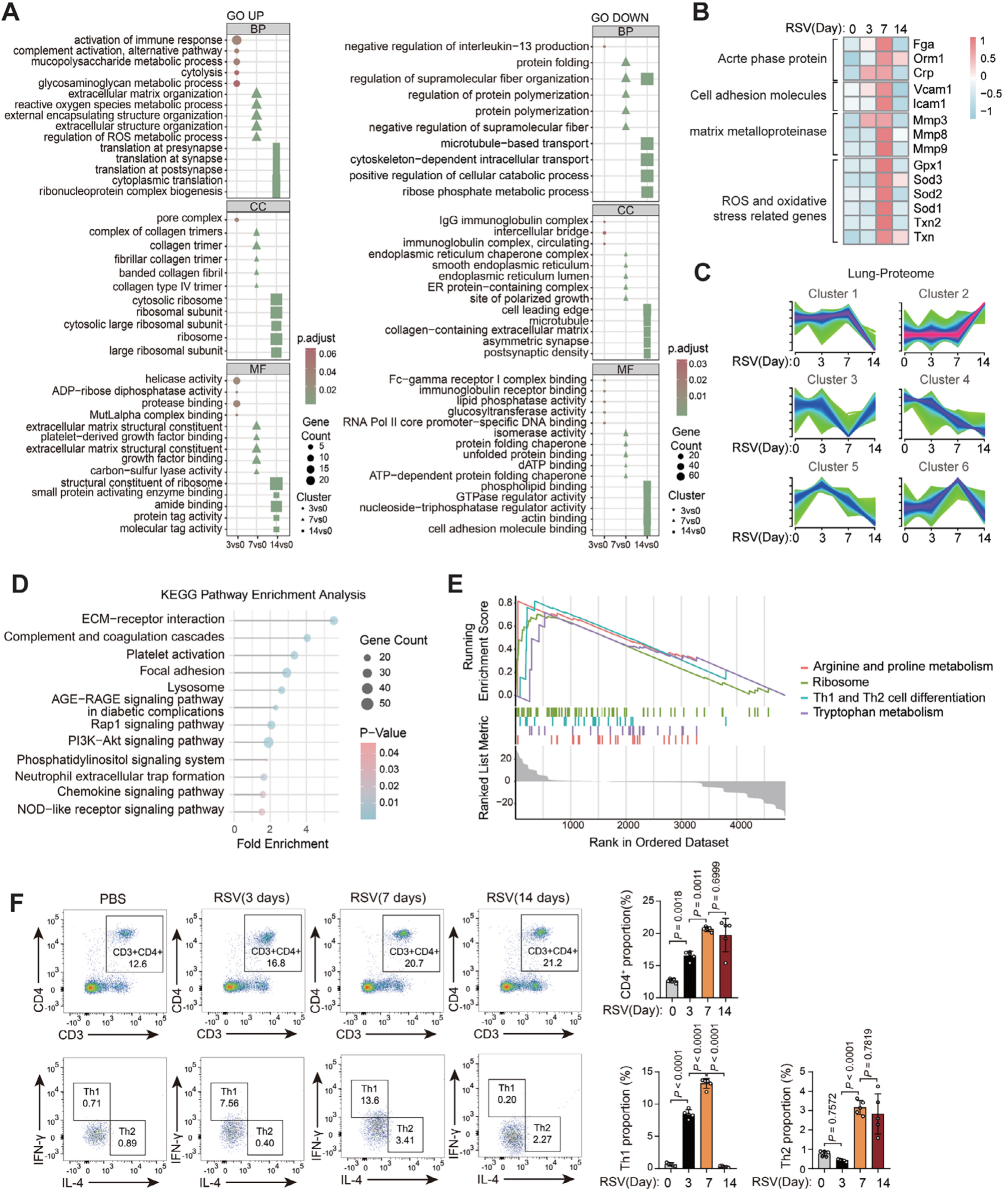
Proteomic characteristics of lung tissue in RSV‐infected mice. (A) GO enrichment analysis of upregulated and downregulated proteins in lung tissues at 3, 7, or 14 dpi compared with 0 dpi (3vs0, 7vs0, and 14vs0). Bubble charts show enriched terms for BP, CC, and MF categories. (B) Heatmap showing expression levels of lung injury‐associated proteins at 0, 3, 7, and 14 dpi. Relative expression is indicated by the color gradient (blue: downregulated; red: upregulated). (C) Fuzzy c‐means clustering analysis of quantified proteomic data, revealing six distinct expression clusters over the infection time course. Data points represent quantitative protein expression from individual mouse lungs at each time point. (D) KEGG pathway enrichment analysis for protein clusters 1 and 6. Bubble size represents the proportion of target proteins enriched in the pathway; color intensity corresponds to statistical significance (red: most significant; blue: least significant). (E) GSEA showing pathways significantly activated during the recovery period (14 dpi vs. 7 dpi). (F) Flow cytometric analysis of Th1 (IFN‐γ^+^ CD4^+^) and Th2 (IL‐4^+^ CD4^+^) T cells in lung single‐cell suspensions from mice at 0, 3, 7, and 14 dpi. Bar graphs depict the proportion of each subset among CD4^+^ T cells. Data are presented as mean ± s.d. (*n* = 5 mice per group). Statistical significance was determined by one‐way ANOVA with Tukey's post‐hoc test.

At 3 dpi, 168 differentially abundant proteins (DAPs) were identified, comprising 138 upregulated and 30 downregulated proteins. Upregulated proteins were primarily involved in biological processes related to complement system activation and innate immunity, indicating an early innate immune defense against viral invasion. Downregulated proteins exhibited marked suppression of antibody‐mediated immune functions, including IgG complex formation and Fcγ receptor binding. Based on the finding that RSV can actively suppress early antibody effector mechanisms to evade Fcγ‐mediated phagocytosis and ADCC clearance [24], we speculate that this suppression phenomenon likely stems from the virus's immune evasion strategy (Figure 3A).

At 7 dpi, a total of 263 DAPs were detected (151 upregulated, 112 downregulated). Upregulated proteins were predominantly enriched in ROS metabolism and ECM‐related processes, findings underscore the significant roles of oxidative stress responses and ECM remolding in immune function, cellular repair, and tissue restructuring during the peak phase of RSV infection. Enhanced ROS production is likely to act as critical signaling mediators in antiviral defense, while also potentially contributing to oxidative tissue damage, a common feature in severe viral pneumonitis [25, 26]. Additionally, ECM‐associated processes suggest the involvement of matrix remodeling and fibrotic responses in coordinating immune and repair activities [27]. Conversely, downregulated processes were strikingly enriched in the regulation of protein folding and supramolecular fiber organization/protein polymerization. This indicates significant disruption in proteostasis and cytoskeletal organization during peak infection, potentially reflecting cellular stress or resource diversion (Figure 3A).

At 14 dpi, 1000 DAPs were identified with 279 upregulated and 721 downregulated. Upregulated biological processes were predominantly linked to ribosomal biogenesis and diverse translational events, a hint of sustained robust protein synthesis during the late infection phase. This activity, occurring alongside a likely dampened immune response, may support immune recovery, preserve core cellular functions, and address the dual demands of tissue repair and viral clearance. In contrast, downregulated pathways were primarily associated with catabolic processes and intracellular trafficking. This suggests a general reduction in metabolic processes late in infection, possibly associated with cellular stress responses and progressive immune suppression. Inhibition of catabolism implies decreased cellular energy production, potentially impacting the ability to sustain basic physiological activities. Such metabolic reprogramming, including dampened catabolism, is a hallmark of immune cell exhaustion in chronic infections and can also occur in the resolution phase of acute inflammation [28]. Reduced intracellular transport may reflect functional decline in compromised cells or the emergence of immune evasion mechanisms (Figure 3A).

Of note, the expression levels of lung injury marker proteins peaked at 7 dpi, a temporal pattern lagged behind their transcriptional peaks observed at 3 dpi (Figure 3B). This disconnection between transcript and protein dynamics is likely arises from the dynamic balance between protein synthesis and degradation. Specifically, although transcription of the injury marker genes peaked early (at 3 dpi), the subsequent translation into protein and the rate of new protein synthesis likely remained sufficiently high and consistently exceeded the rate of protein degradation over the following days. This sustained net positive balance (synthesis > degradation) allowed the proteins to progressively accumulate, ultimately reaching their peak abundance later, at 7 dpi.

Fuzzy c‐means clustering of dynamic proteomic profiles partitioned proteins into six clusters (Figures 3C and S2). Among these, Cluster 1 and Cluster 6 exhibited expression trajectories concordant with injury marker proteins. Enriched pathways within these clusters exhibited substantial overlap with those identified in the transcriptome analysis. Among these were inflammation‐ and immune‐associated pathways, including the complement system, cytokine signaling, and NOD‐like receptor signaling. Alongside, processes related to ECM remodeling, as well as the PI3K–AKT and Rap1 signaling pathways were also enriched (Figure 3D).

The interval from 7 to 14 dpi corresponds to the lung injury recovery phase, during which proteins with marked expression changes are presumably pivotal to tissue repair. To dissect the underlying molecular events, we conducted Gene Set Enrichment Analysis (GSEA) on the lung proteome data (Figure 3E). The GSEA results suggest that core features of recovery involve key upregulated pathways centered on tissue reconstruction and immune coordination, prominently including enhanced amino acid metabolism, ribosomal function, and Th1/Th2 cell differentiation. Based on the well‐established role of enhanced amino acid metabolism in supporting cellular effector functions and repair processes [29, 30], we speculate that the observed enhancement likely serves to fuel tissue regeneration and immune cell proliferation. The significant increase in ribosome biogenesis and function, the rate‐limiting step in protein synthesis, strongly implies an elevated demand for protein production essential for tissue repair. This integrated metabolic and translational shift appears fundamental to the recovery phase.

To functionally validate the involvement of T helper cell subsets, we performed flow cytometry to directly quantify Th1 and Th2 cells in the lungs throughout RSV infection. The proportions of both Th1 (IFN‑γ^+^ CD4^+^) and Th2 (IL‑4^+^ CD4^+^) T cells were low in uninfected lungs (0 dpi). At 3 dpi, we observed a significant increase in Th1 cells, while Th2 cells remained at baseline levels, indicating an early bias toward a proinflammatory Th1 response. By the peak injury phase (7 dpi), the Th1 population continued to expand, and a concurrent rise in Th2 cells became evident, suggesting the initiation of a counter‑regulatory or repair‑oriented Th2 response alongside persistent inflammation. Strikingly, during the resolution and repair phase at 14 dpi, the proportion of Th1 cells dropped markedly to near preinfection levels. In contrast, Th2 cells showed a more sustained presence, with only a modest decline from their peak at 7 dpi (Figure 3F). These data provide direct cellular evidence for a dynamic shift from a dominant Th1‑driven inflammatory environment during acute injury to a Th2‑skewed milieu in the repair phase, suggested a key role of adaptive immunity in shaping the recovery microenvironment.

### 2.4 Proteomic Feature in Human Lung Bronchial Organoid With RSV Infection

Compared with mouse models with inherent species differences, human lung bronchial organoids better replicate human organ structure, function, and physiological conditions. We therefore employed a human lung bronchial organoid model. Organoids were sampled at 0, 3, 7, and 14 dpi and subjected to comprehensive proteomic analysis. A total of 3423 proteins were identified, with proteomic profiles demonstrating significant temporal variation.

**FIGURE 4:**
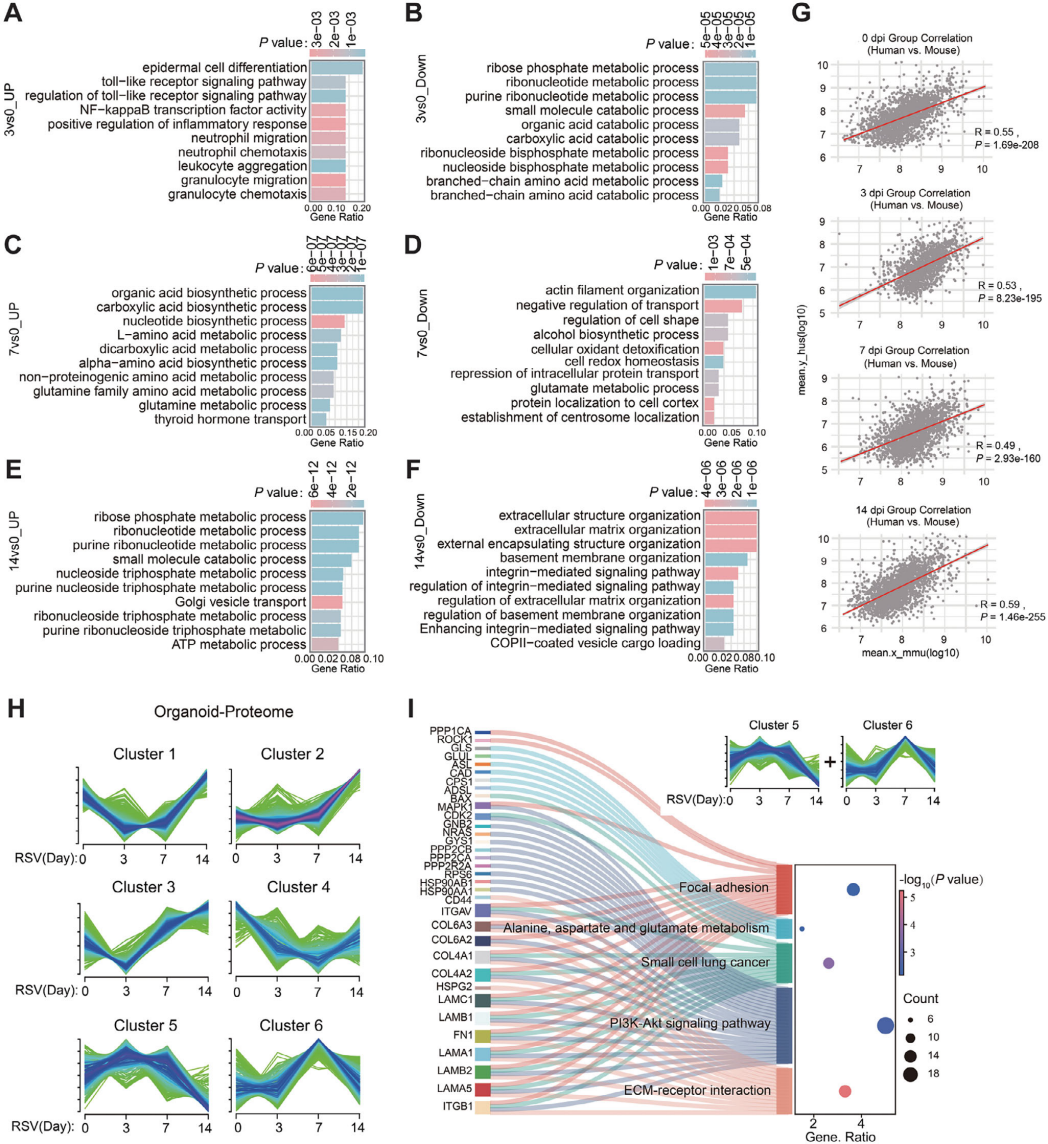
Proteomic features of human lung bronchial organoids following RSV infection. (A–F) Bar charts showing Gene Ontology Biological Process (GO‐BP) enrichment analysis of differentially expressed proteins in human lung bronchial organoids at 3, 7, and 14 dpi verse 0 dpi (3vs0, 7vs0, and 14vs0). Panels (A, C, E) show enrichment of upregulated proteins; panels (B, D, F) show enrichment for downregulated proteins. (G) Scatter plot depicting the proteomic correlation between human bronchial organoids and mouse lung tissue (Spearman's correlation). (H) Fuzzy c‐means clustering analysis of quantified proteomic data, revealing six distinct expression clusters over the infection time course. Data points represent quantitative protein expression from individual human lung bronchial organoids at each time point. (I) Sankey diagram (left) and bubble chart (right) representing correlation patterns derived from network analysis. The Sankey diagram visualizes associations between lung injury‐related gene clusters (5 and 6, identified by Fuzzy c‐means analysis in (H)) and specific biological pathways (colored lines). The bubble chart shows KEGG pathway enrichment for the associated genes; bubble size represents the proportion of enriched target genes, and color intensity indicates statistical significance (red: most significant, low *p* value; blue: least significant, high *p* value).

In the early stage postinfection (3 dpi), human organoids exhibited a robust innate immune response, characterized by significant upregulation of the TLR signaling pathway and NF‐κB activation (Figure 4A). This aligns with the early immune defense response observed in mouse models. Concurrently, numerous metabolic pathways were broadly suppressed (Figure 4B).

Transitioning to the intermediate stage (7 dpi), a shift in focus occurred: biosynthesis and metabolic pathways involving fundamental building blocks, such as amino acids and nucleotides, became highly active (Figure 4C). This partially overlaps with the phenomenon in mice where resources are mobilized for repair and stress responses (e.g., production of ECM or ROS). Intriguingly, a key divergence emerged: pathways responsible for maintaining cellular chemical balance (redox homeostasis) and detoxifying harmful oxidative species were downregulated in human organoids (Figure 4D). This contrasts sharply with the typical increase in ROS seen in mice at this time point, highlighting a difference in stress‐handling mechanisms between the two models.

By the late stage (14 dpi), the differences between human organoids and mouse models became more pronounced. In human organoids, catabolic processes responsible for breaking down substances to obtain energy were significantly upregulated (Figure 4E). This presents a stark contrast to the pattern in mice during the recovery phase, where catabolism is suppressed and protein synthesis is enhanced. Furthermore, human organoids displayed unique alterations in cell interactions with their surrounding structural support, characterized by downregulation of integrin signaling and ECM regulatory pathways (Figure 4F). In contrast, late‐stage changes in mice primarily involved the suppression of global protein homeostasis.

To quantify the similarity in proteomic response patterns between the human bronchial organoid model and the murine lung tissue model following RSV infection, we performed Pearson correlation coefficient analysis on the two proteomic datasets. The results showed moderate to strong positive correlations (*R* > 0.5) at all four time points: *R* = 0.55 (0 dpi), *R* = 0.53 (3 dpi), *R* = 0.51 (7 dpi), and *R* = 0.60 (14 dpi) (Figure 4G). These significant correlations indicate a substantial concordance in the overall trends of RSV‐induced proteomic changes between the two systems. Importantly, given that human bronchial organoids represent a simplified in vitro model and cannot fully recapitulate the complexity of an intact in vivo lung tissue, especially the murine model encompassing multiple cell types, immune components, and systemic factors, the observed moderate‐to‐strong correlations are notably high.

Further analysis of dynamic proteomic patterns in organoids identified Cluster 5 and Cluster 6 as exhibiting expression trajectories similar to the progression of lung tissue injury (Figures 4H and S3). Pathway enrichment analysis of DAPs within these clusters revealed significant enrichment for pathways including PI3K–Akt signaling and ECM‐receptor interaction (Figure 4I). This finding demonstrates high consistency with the results from the murine lung tissue proteomic analysis.

### 2.5 Integrated Multiomics Analysis Reveals an Integrin‐Centric Network Driving RSV‐Induced Lung Injury and Identifies Diagnostic Biomarkers

**FIGURE 5:**
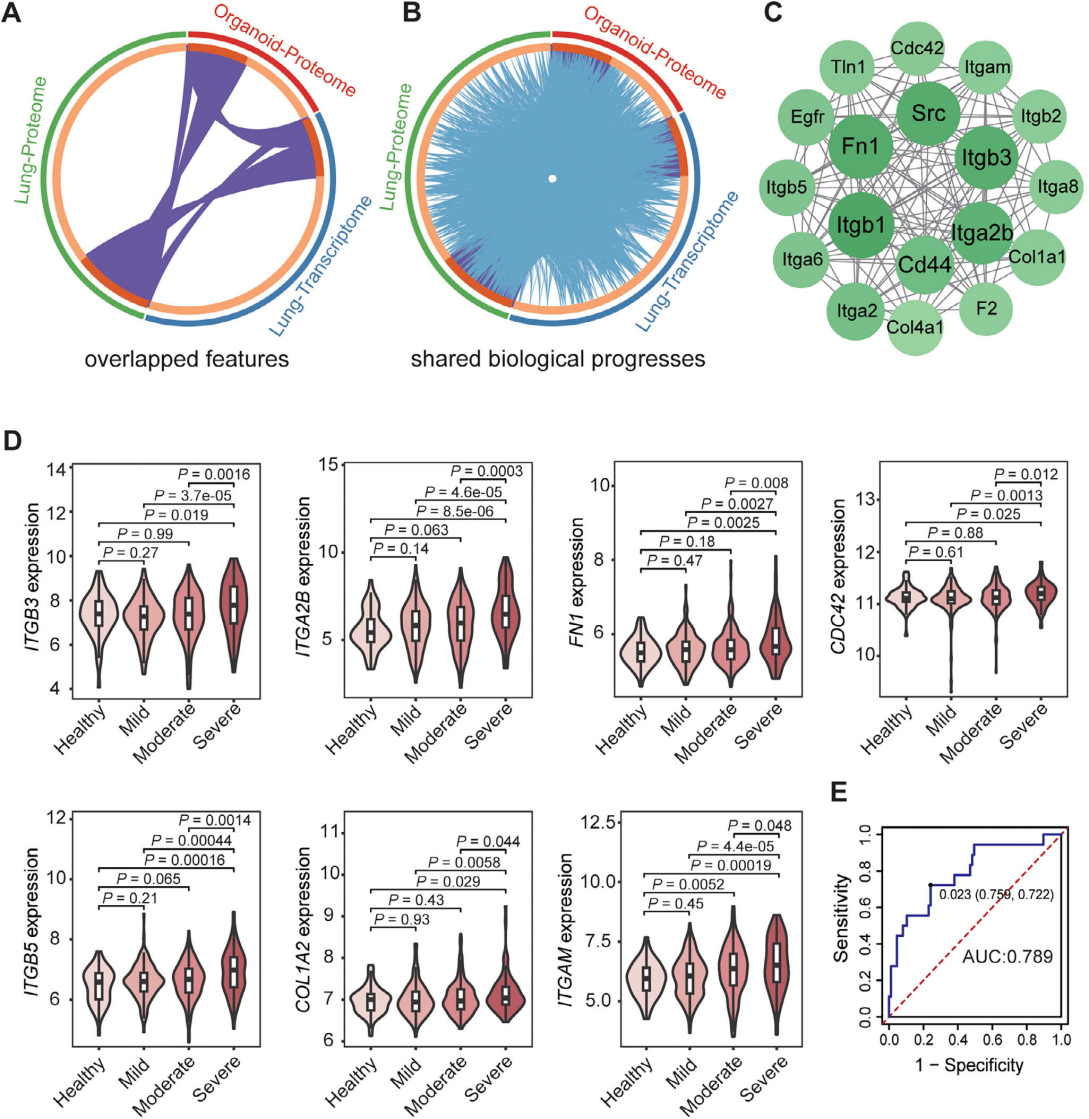
Integrated multiomics analysis identifies key genes and assesses their clinical value. (A) Overlap of lung injury‐associated gene/protein sets across multiomics datasets. The Circos plot visualizes the compositional overlap between six gene/protein clusters derived from: transcriptomic clusters 3 and 4 (Lung‐Transcriptome), proteomic clusters 1 and 6 (Lung‐Proteome), and proteomic clusters 5 and 6 (Organoid‐Proteome). Connections represent shared genes/proteins among clusters (generated using Metascape). (B) Overlap of enriched biological processes associated with the multiomics gene/protein sets. The Circos plot illustrates the similarity in enrichedGO‐BP terms among the six gene/protein clusters defined in (A). Connections represent shared significantly enriched GO‐BP terms, highlighting common functional themes across the clusters (generated using Metascape). (C) PPI network of key genes constructed using the STRING database and visualized in Cytoscape. Core genes (hubs) were identified using the Degree algorithm. Node color intensity (green) corresponds to network importance (darker green means higher degree centrality). (D) Hub gene expression in PBMCs from RSV‐infected infants, analyzed using GEO dataset GSE246622. mRNA expression levels of the hub genes identified in (C) are shown. Sample sizes: healthy controls (*n* = 56), mild (*n* = 156), moderate (*n* = 213), severe (*n* = 96). *p* Values were determined by two‐sided Wilcoxon test. (E) Evaluation of the XGBoost (eXtreme Gradient Boosting) predictive model. The ROC curve demonstrates the model's performance in accurately predicting severe RSV cases within the clinical cohort (AUC = 0.789).

To achieve a comprehensive understanding of the progression of RSV‐induced lung injury, we conducted an integrated analysis of injury progression‐related clusters from the three omics datasets using Metascape. The results revealed minimal overlap in the specific genes/proteins identified between clusters from these datasets (Figure 5A), while the biological processes associated with these clusters exhibited substantial convergence (Figure 5B). To further clarify how these shared biological terms related to one another, we visualized a subset of them as an interaction network (Figure S4A). This network explicitly illustrates strong interconnections between the terms, pointing to a high probability of their coordinated regulation of lung injury and repair processes.

To identify potential biomarkers and therapeutic targets for RSV‐induced lung injury, we constructed a PPI network for key genes involved in the aforementioned processes using the STRING database. Subsequently, we employed the MCODE plugin within Cytoscape to score the network based on connectivity and visualized the PPI network according to these scores (Figures 5C and S4B). This analysis ultimately identified five primary hub genes (*Itgb1*, *Itgb3*, *Itga2b*, *Fn1*, *Src*) and 12 secondary hub genes (*Cdc42*, *Itgam*, *Itgb2*, *Itga8*, *Col1a2*, *F2*, *Cd44*, *Itga1*, *Itga*, *Itgb5*, *Egfr*, *Tln1*).

The construction of a PPI network identified key hub genes predominantly belonging to the integrin family (*Itgb1* (CD29), *Itgb3* (CD61), *Itga2b* (ITA2B), *Itgam* (CD11b), *Itgb2* (CD18), *Itga8* (ITA8), *Itga1* (ITA1), *Itgb5* (ITB1)), ECM components (*Fn1* (fibronectin), *Col1a2* (CO1A2)), and signaling molecules (*Src* (SRC), *Cdc42* (CDC42), *Tln1* (TLN1), *F2* (prothrombin), *Cd44* (CD44), *Egfr* (EGFR)). These hubs form a cohesive network orchestrating multiple interconnected pathophysiological processes underlying RSV‐induced lung injury: (1) leukocyte adhesion and migration, mediated by integrins (e.g., CD11b/ CD18), facilitating immune cell extravasation and activation within inflamed tissue [31, 32, 33]; (2) platelet activation and thrombosis, driven by αIIbβ3 (encoded by *Itga2b* and *Itgb3*) to promote platelet aggregation and vascular damage [34, 35]; (3) ECM dysregulation and fibrosis, characterized by aberrant fibronectin and CO1A2 deposition regulated through integrin signaling (e.g., CD29), leading to pathological tissue remodeling [36, 37, 38, 39, 40]; (4) cytoskeletal signaling and cell motility, transduced by SRC, CDC42, and TLN1 in response to integrin and growth factor signals, governing cell adhesion, migration, and repair processes [41, 42, 43]; (5) coagulation and inflammation, where prothrombin acts via PARs to induce vascular permeability, inflammation, and fibrosis [44, 45]; and (6) DAMP signaling and repair, involving CD44 binding damage‐associated molecular patterns (e.g., HA fragments) to modulate leukocyte behavior and tissue repair [46]. Collectively, this network analysis implicates dysregulated cell–ECM interactions, adhesion signaling cascades, and coagulation pathways as the central mechanistic drivers in RSV pathogenesis.

To explore the clinical relevance of these hub genes, we examined their expression in a GEO dataset (GSE246622) comprising peripheral blood transcriptomes from healthy infants and patients with mild, moderate, or severe RSV infection. Among the identified key genes, *Itgb3*, *Itga2b*, *Fn1*, *Cdc42*, *Itgam*, *Col1a2*, and *Itgb5* showed significant positive correlations with disease severity (Figure 5D). Next, we employed an XGBoost model to evaluate the clinical utility of these seven genes. The model achieved an area under the curve (AUC) of 0.789 in distinguishing severe from non‐severe patients, highlighting their robust clinical predictive value (Figure 5E).

### 2.6 Functional Validation of the Integrin–Fn1 Axis as a Driver of RSV‐Induced Lung Injury and Repair

**FIGURE 6:**
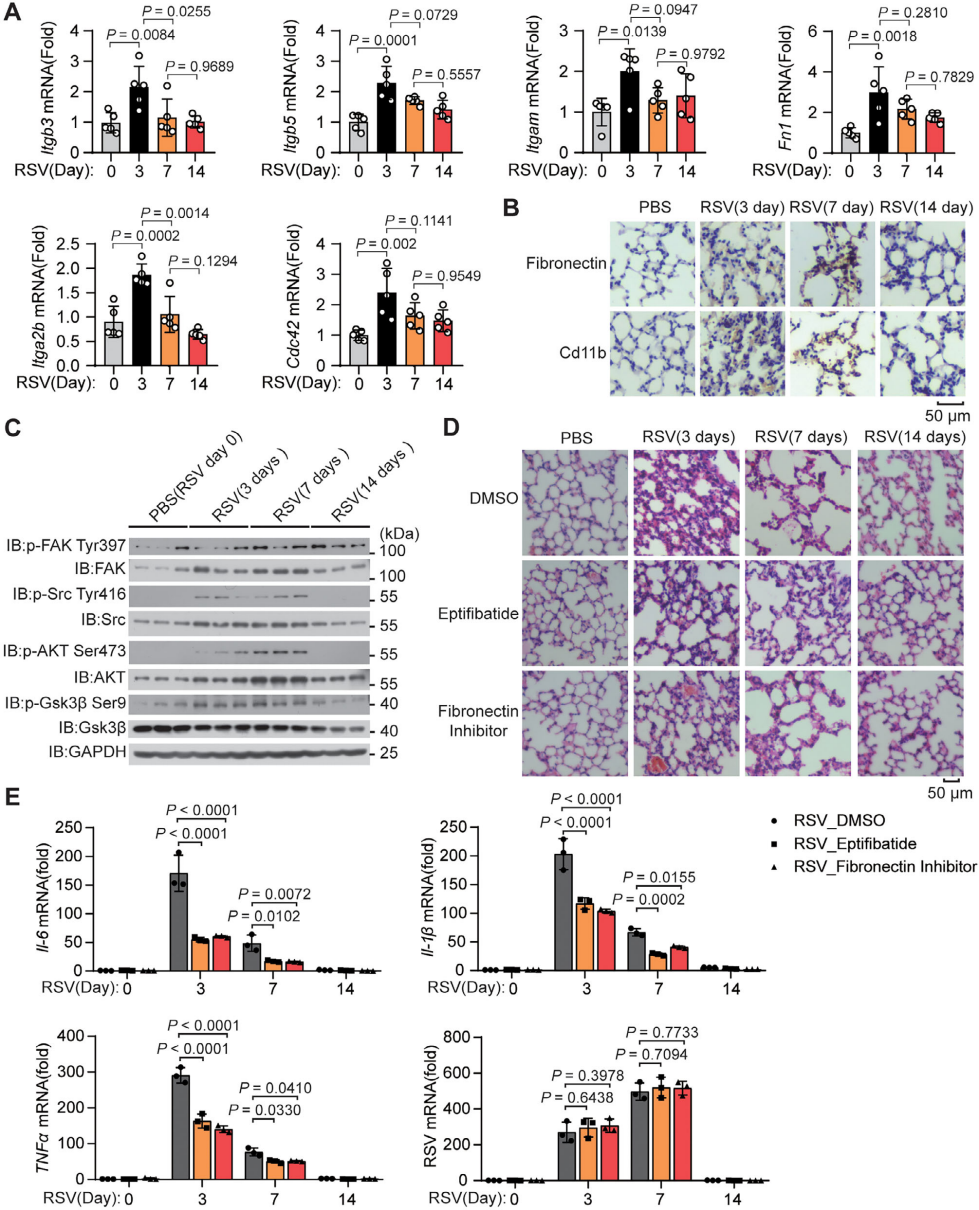
Functional validation of the integrin–Fn1 axis as a driver of RSV‐induced lung injury and repair. (A) qPCR validation of mRNA expression for the core genes (*Itgb3*, *Itgb5*, *Itgam*, *Fn1*, *Itga2b*, *Cdc42*) identified in (C) in mouse lung tissues. *n* = 5 per group. Statistical analysis was performed using two‐way ANOVA. (B) Immunohistochemical (IHC) staining of fibronectin (*Fn1*) and CD11b (*Itgam*) protein expression in mouse lung tissue at 0, 3, 7, and 14 dpi. Scale bars, 50 µm. (C) Immunoblot analysis of the expression and phosphorylation levels of key signaling proteins in lung tissue lysates from mice at 0/3/7/14 dpi. The blots were probed with antibodies against the phosphorylated and total forms of FAK (Tyr397), Src (Tyr416), AKT (Ser473), and GSK3β (Ser9). GAPDH was used as a loading control. (D) H&E‐stained lung sections from DMSO/eptifibatide/fibronectin inhibitor‐treated mice at 0/3/7/14 dpi. Scale bars, 50 µm. (E) qPCR analysis of *Il6*, *Il1b*, *Tnf*, and RSV mRNA in lungs of DMSO/eptifibatide/fibronectin inhibitor‐treated mice at 0/3/7/14 dpi. *n* = 3 per group. Statistical analysis was performed using two‐way ANOVA.

To validate the reliability of the identified hub genes, we first examined their expression dynamics in mouse lung tissue. qPCR confirmed that *Itgb3*, *Itgb5*, *Itgam*, *Fn1*, *Itga2b*, and *Cdc42* transcripts peaked at 3 dpi and subsequently declined, consistent with the transcriptomic findings (Figure 6A). Immunohistochemical staining for fibronectin (*Fn1*) and CD11b (*Itgam*). revealed that both proteins peaked at 7 dpi, in line with the proteomic injury marker dynamics (Figure 6B).

Although the PPI network predicted physical interactions among these hub genes, whether the integrin‐centered signaling axis is functionally activated during RSV infection remained to be determined. Canonical integrin signaling mechanisms indicate that integrin ligation triggers assembly of the FAK/Src signaling complex, which further activates the PI3K–AKT pathway and its downstream effector GSK3β [47‐50]. Accordingly, we detected the phosphorylation levels of core downstream signaling molecules. Western blot analysis showed that p‐FAK (Tyr397), p‐Src (Tyr416), p‐AKT (Ser473), and p‐GSK3β (Ser9) all exhibited a coordinated increase, peaking at 7 dpi before declining at 14 dpi (Figure 6C). This dynamic profile indicates that integrin engagement is followed by sequential activation of FAK/Src and the downstream PI3K–AKT/GSK3β cascade, functionally linking the upregulated network components to pathways governing cell adhesion, survival, and inflammatory/fibrotic responses.

To establish a direct causal role, we performed in vivo pharmacological intervention studies. Mice were treated with either eptifibatide (an integrin αIIbβ3 inhibitor) or a fibronectin inhibitory peptide prior to and during RSV infection. Histopathological analysis at 7 dpi revealed that both inhibitors significantly attenuated lung damage, as evidenced by reduced inflammatory infiltration, alveolar wall thickening, and congestion (Figure 6D). Consistent with this, the pulmonary upregulation of *Il6*, *Il1b*, and *Tnf* was markedly suppressed in inhibitor‐treated groups. Notably, neither inhibitor affected viral load (Figure 6E), indicating that the protective effect resulted from mitigation of the dysregulated host response rather than altered viral replication. These in vivo interventions demonstrate that targeting αIIbβ3 or fibronectin effectively reduces immunopathology without compromising antiviral defense, functionally validating their critical role in RSV pathogenesis and underscoring their therapeutic potential.

## 3 Discussion

This study presents a comprehensive multiomics landscape of the dynamic host response to RSV infection, spanning from acute lung injury to resolution and repair. By integrating longitudinal transcriptomic and proteomic profiling of murine lung tissue and human bronchial organoids with in vivo functional validation, we identify a dysregulated integrin–ECM signaling axis as a central, causal driver of RSV‐induced immunopathology. This core network, centered on *Itgb3*, *Itga2b*, and *Fn1*, was consistently enriched across models and omics layers. Expression levels of these integrin–ECM‐related genes correlated with disease severity in independent clinical cohorts and demonstrated robust predictive performance in machine learning models. Pharmacological inhibition of these genes significantly attenuated lung injury and inflammation, indicating their roles in clinical prediction and therapy.

Integrins are transmembrane receptors that mediate cell–ECM adhesion and bidirectional signaling, playing fundamental roles in tissue homeostasis, immune cell trafficking, wound healing, and fibrosis [51, 52]. Upon tissue injury, integrins orchestrate core pathological processes such as immune cell recruitment, hemostasis and thrombosis, and ECM remodeling, which collectively drive inflammation, tissue repair, and fibrotic responses [53, 54, 55, 56, 57]. Dysregulation of the integrin–ECM axis has been implicated in acute lung injury, pulmonary fibrosis, and viral pneumonia, such as the one that induced by influenza viruses or SARS‐CoV‐2 [31, 58, 59, 60]. However, the specific integrin heterodimers and downstream signaling pathways underlying virus‐induced immunopathology have not been systematically defined. In this study, multiomics analysis across murine lungs and human bronchial organoids showed that the integrin–ECM network centered on *Itgb3*, *Itga2b*, and *Fn1* is pivotally dysregulated during RSV‐induced lung injury. This positions the integrin–ECM axis as a central, therapeutically targetable hub in RSV‐induced immunopathology. However, as cell‐type‐specific contributions to integrin–ECM cascade were still obscure, future studies employing single‐cell or spatial transcriptomics or proteomics will be critical to resolve which cellular populations (e.g., alveolar epithelium, recruited macrophages, fibroblasts) are the primary drivers of this integrin–ECM cascade and inform more precise therapeutic strategies.

Our multiomics data also reveal a pronounced early suppression of genes associated with humoral immunity and antibody effector functions (e.g., immunoglobulin production, Fcγ receptor binding) at both transcriptomic and proteomic levels. This coordinated downregulation is consistent with known RSV immune evasion strategies—including NS1/NS2‐mediated interference with type I IFN signaling and germinal center responses [24, 61]—and provides systematic molecular evidence suggesting that RSV may actively create a window to evade Fc‐dependent clearance from the outset of infection. We hypothesize that this early suppression delays high‐affinity neutralizing antibody responses, shifting the burden of viral control toward innate immunity and delayed CTL activity. This shift may have dual consequences: it extends the viral replication window (consistent with the delayed viral peak at 7 dpi) and exacerbates immunopathology, as persistent virus and a dysregulated CTL response coincide with maximal tissue injury. Furthermore, this early immune reprogramming may influence the repair phase by altering the subsequent Th1/Th2 balance. Our data show a sustained Th2 presence during repair (14 dpi), which is necessary for tissue restoration but may promote fibrosis if dysregulated [62, 63, 64]. Thus, early immune suppression may not only shape acute disease severity but also determine the quality of long‐term lung repair. This framework offers a mechanistic rationale for early immune interventions—such as enhancing mucosal antibody responses or passive immunization—to prevent severe RSV disease and its long‐term pulmonary consequences.

Another key temporal feature is the uncoupling between peak injury‐related gene transcription (3 dpi) and peak protein abundance (7 dpi), indicating a dynamic, multilayered regulatory program rather than technical variation. To explain this, we propose an integrated model of regulated translation and protein stabilization: at 3 dpi, potent innate immune signals (e.g., NF‐κB) drive a rapid transcriptional burst, creating a reservoir of mRNA templates; however, de novo translation may be initially restrained by stress‐responsive pathways or specific posttranscriptional controls (e.g., via miRNAs implicated in inflammation) to prevent premature and potentially detrimental protein accumulation [65, 66, 67]. As the infection progresses to the peak injury phase (7 dpi), context‐dependent signals from the evolving microenvironment, including sustained integrin activation and oxidative stress, trigger a coordinated shift that relieves translational repression and enhances the stability of newly synthesized proteins [68, 69] (e.g., by modulating ubiquitin–proteasome activity). This is supported by our proteomic data (Figure 3E; GSEA), which reveal significant enrichment of translational machinery and protein stability pathways at this stage. This staggered pattern enables the host to produce inflammatory proteins first, while delaying tissue‐remodeling factors until the peak phase of injury and repair. As such, the observed delay between transcript and protein peaks may reflect an active, adaptive mechanism, suggesting that future therapies could target not only gene transcription but also this regulatory juncture.

Organoids recapitulate key structures and functions of native tissues and act as reliable physiological models for studying human development, disease mechanisms, and drug responses. Still, there existed substantial differences between human bronchial organoids and murine lungs in our study. First, unlike intact murine lungs, organoids lack circulating immune cells, vasculature, and systemic signals [70], which accounts for the absence of complex immune pathways such as the complement and coagulation cascades. Second, metabolic disparities exist, such as upregulated catabolism in late‐stage organoids versus catabolic suppression in murine lungs. This disparity likely stems from fundamental differences in energy utilization strategies: systemically supported pulmonary tissue can harness external resources for anabolic repair, whereas isolated epithelial cultures in defined culture medium must undergo cell‐autonomous catabolic adaptation. Of note, although these model‐specific variations exist, core injury‐associated signaling networks, specifically PI3K–Akt signaling and ECM–integrin interactions, were consistently enriched in both systems, demonstrating conservation of the epithelium‐intrinsic pathological core. Thus, while organoids cannot fully recapitulate the systemic immune landscape of lung injury, they faithfully capture local epithelial responses that occur in the complex in vivo microenvironment.

Of note, the hub genes we identified in mouse lung tissues and human bronchial organoids could reflect the clinical severity of RSV infection, as determined from peripheral blood. This association underscores the potential of peripheral blood markers to reflect lung pathology, likely through a systemic inflammatory “echo”: local lung inflammation releases signals that enter the circulation, mobilizing integrin‐expressing immune cells (e.g., monocytes, platelets) from the bone marrow and periphery into the blood, where their upregulated integrin transcript levels become detectable prior to or during recruitment into the infected lung. While this provides a rationale for using blood‐based biomarkers, further studies are needed to directly validate the mechanistic link between circulating integrin signatures and local pulmonary pathology.

Several limitations of this study should be acknowledged. First, our transcriptomic and proteomic analyses were conducted using whole lung tissue or organoid homogenates, which limits the ability to identify cell‐type‐specific contributions. The integrin–ECM signaling network identified in this study may be differentially activated in epithelial, endothelial, immune, and fibroblast populations. Future studies using single‐cell or spatial omics technologies are needed to clarify the precise cellular sources of this pathological cascade. Second, although our in vivo inhibitor assays and Western blot analyses have verified the causal role of the integrin–Fn1 axis and its downstream FAK/Src/PI3K–Akt signaling pathway, the detailed molecular mechanisms remain to be fully elucidated.

In conclusion, this study not only describe multiomics atlas of RSV‐induced lung injury, but also deliver a mechanistically validated, translationally anchored, and clinically correlated understanding of this progress, implying a hint for host‐directed interventions for severe RSV disease in the future.

## 4 Methods

### 4.1 Animal Model Construction

Six‐week‐old male C57BL/6J mice were purchased from Shanghai Jihui Laboratory Animal Technology Co., LTD. Mice were housed in a specific pathogen‐free facility at Soochow University. After anesthesia with 10% chloral hydrate, mice were intranasally infected with 1 × 10^8^ PFU RSV, while control mice received an equal volume of PBS. Animals were euthanized at 0, 3, 7, and 14 dpi. For inhibitor treatment, mice were randomly divided into DMSO, eptifibatide (2.5 mg/kg), or fibronectin inhibitor (10 mg/kg) groups. Drugs or DMSO were administered daily by oral gavage from 1 day before infection until the day before sacrifice. All virus‐related experiments were performed in a P2 laboratory.

### 4.2 Organoids

Human bronchial organoids were kindly provided by Prof. Bing Zhao (Fudan University). Organoids were cultured in Matrigel‐coated 24‐well plates with DMEM/F12 medium supplemented with 10% FBS at 37°C in a 5% CO_2_ atmosphere.

### 4.3 RNA‐seq and Proteomic Analyses

Total RNA was extracted from lung tissues and used for library construction and sequencing on an Illumina Novaseq 6000 platform. Proteomic and phosphoproteomic analyses were performed using an Orbitrap Fusion Lumos mass spectrometer. Detailed procedures are provided in the Supporting Information: Materials and Methods.

### 4.4 Bioinformatics Analysis

Differential expression analysis, GO/KEGG enrichment, GSEA, immune infiltration analysis, time‐series clustering, and PPI network analysis were performed using standard bioinformatic pipelines. Detailed parameters and software versions are listed in the Supporting Information: Materials and Methods.

### 4.5 XGBoost Model Analysis

The clinical significance of hub genes was verified using the GEO dataset GSE246622 and an XGBoost classification model.

### 4.6 QPCR, Immunoblot, Histopathology, IHC, and Flow Cytometry

Quantitative real‐time PCR, immunoblotting, hematoxylin and eosin staining, immunohistochemistry, and flow cytometry were performed according to standard protocols. Primer sequences, antibodies, and detailed procedures are provided in the Supporting Information: Materials and Methods.

### 4.7 Statistical Analysis

For omics data, a false discovery rate‐adjusted *p* value < 0.05 was considered significant. For other experiments, data are presented as mean ± SD. Comparisons between two groups were performed using two‐tailed unpaired Student's *t*‐test, and multiple groups were analyzed by two‐way ANOVA. A *p* value < 0.05 was considered statistically significant.

## Author Contributions

Lili Zhou performed most experiments and drafted the original manuscript. Hua Guo established and validated mouse RSV infection models and performed mass spectrometry experiments. Xiaofeng Yu, Ruiqi Liu, and Ran Li conducted the bioinformatics analysis of the multiomics data. Yu Ran provided guidance for mass spectrometry experiments. Jiali Zhu performed culture and maintenance of human bronchial organoids. Leshi Chen and Yongliang Zhu provided guidance for human bronchial organoid experiments. Long Zhang, Zhenjiang Bai, and Jie Xu reviewed the manuscript. Fangfang Zhou supervised the study, designed the experiments, and revised the manuscript. All authors critically reviewed and approved the final manuscript.

## Funding

This work was supported by the National Key R&D Program of China (grant no. 2022YFA1105200 to F.Z.), the National Science Fund for Distinguished Young Scholars (grant no. 32125016 to F.Z.), the Chinese National Natural Science Funds (grant no. U24A20371 to F.Z., 32525002, W2411011 to L.Z. and T2321005 to Z.L.), Suzhou Medical College‐QiLu Medical Research Program of Soochow University (grant no. 24QL101301 to F.Z.), the Priority Academic Program Development of Jiangsu Higher Education Institutions, and the Natural Science Foundation of Jiangsu Province (No. BK20255001).

## Conflicts of Interest

The authors declare no conflicts of interest.

## Ethics Statement

Animal experiments were approved by the Animal Ethics Committee of Soochow University (Approval No. SUDA20260521A01).

## Supporting information




**Supporting Figure 1**: Fuzzy c‐means clustering reveals temporal gene expression patterns in RSV ‐induced lung injury. At each time point, the quantitative mRNA data from multiple mice were classified into six different expression clusters using the fuzzy c‐means clustering method, in order to demonstrate the relative transcriptional expression changes in the mouse model infected with RSV. The pathways corresponding to this cluster of genes obtained through KEGG enrichment analysis are on the right side.
**Supporting Figure 2**: Fuzzy c‐means clustering reveals temporal protein expression patterns in RSV‐induced lung injury. Fuzzy c‐means clustering of quantified proteomic data from multiple mice per time point (0, 3, 7, 14 dpi) identified six expression clusters, showing relative translational expression alterations in RSV‐infected mice. Corresponding pathways from KEGG enrichment analysis are on the right.
**Supporting Fig 3**: Fuzzy c‐means clustering of protein expression in RSV‐infected human bronchial organoids. By performing fuzzy c‐means clustering on the quantitative proteomic data obtained from infected human lung bronchial organoids at each time point (0, 3, 7, and 14 days after RSV infection), six expression clusters were identified, revealing the relative translation expression changes in RSV‐infected human lung bronchial organoids. The relevant pathways obtained from KEGG enrichment analysis are shown on the right side.
**Supporting Fig 4**: Functional and network analysis of lung injury‐associated multiomics signatures. (A) Enrichment network of lung injury‐related features across multiomics datasets. Circular nodes represent significantly enriched biological processes (color‐coded by functional category). Elliptical nodes denote signaling pathways (distinct colors indicate different pathways). Analyzed clusters include: transcriptomic clusters 3 and 4 (lung‐transcriptome), proteomic clusters 1 and 6 (lung‐proteome), and proteomic clusters 5 and 6 (organoid‐proteome)—totaling six gene clusters (generated using Metascape). (B) Protein–protein interaction (PPI) network of hub genes driving lung injury pathogenesis. Node color intensity correlates with degree centrality (darker hues means higher connectivity), reflecting key topological roles in the network.

## Data Availability

The transcriptomics data generated in this study have been deposited in the Genome Sequence Archive at the China National Center for Bioinformation (CNCB‐NGDC) under the accession number CRA039516. The mass spectrometry proteomics data have been deposited in the OMIX database under accession number OMIX015436.
