# Supplementary material for: Multiomics Identification and Validation of an Integrin–Extracellular Matrix Network Driving Respiratory Syncytial Virus‐Induced Lung Injury and Repair
Source: MedComm (2020). 2026 Jul 1;7(7):e70811. doi: 10.1002/mco2.70811 (PMC13322079; doi:10.1002/mco2.70811)
Supplement: Supplementary file 1 — Supporting Figure 1: Fuzzy c‐means clustering reveals temporal gene expression patterns in RSV ‐induced lung injury. At each time point, the quantitative mRNA data from multiple mice were classified into six different expression clusters using the fuzzy c‐means clustering method, in order to demonstrate the relative transcriptional expression changes in the mouse model infected with RSV. The pathways corresponding to this cluster of genes obtained through KEGG enrichment analysis are on the right side. Supporting Figure 2: Fuzzy c‐means clustering reveals temporal protein expression patterns in RSV‐induced lung injury. Fuzzy c‐means clustering of quantified proteomic data from multiple mice per time point (0, 3, 7, 14 dpi) identified six expression clusters, showing relative translational expression alterations in RSV‐infected mice. Corresponding pathways from KEGG enrichment analysis are on the right. Supporting Fig 3: Fuzzy c‐means clustering of protein expression in RSV‐infected human bronchial organoids. By performing fuzzy c‐means clustering on the quantitative proteomic data obtained from infected human lung bronchial organoids at each time point (0, 3, 7, and 14 days after RSV infection), six expression clusters were identified, revealing the relative translation expression changes in RSV‐infected human lung bronchial organoids. The relevant pathways obtained from KEGG enrichment analysis are shown on the right side. Supporting Fig 4: Functional and network analysis of lung injury‐associated multiomics signatures. (A) Enrichment network of lung injury‐related features across multiomics datasets. Circular nodes represent significantly enriched biological processes (color‐coded by functional category). Elliptical nodes denote signaling pathways (distinct colors indicate different pathways). Analyzed clusters include: transcriptomic clusters 3 and 4 (lung‐transcriptome), proteomic clusters 1 and 6 (lung‐proteome), and proteomic clusters 5 and 6 (organoid‐proteom [file MCO2-7-e70811-s001.pdf]

Multi-omics identification and validation of an integrin- extracellular matrix (ECM) network driving Respiratory syncytial virus (RSV)-induced lung injury and repair

Lili Zhou[1 2]#, Hua Guo[2]#, Xiaofeng Yu[2]#, Yu Ran[2], Ruiqi Liu[2], Jiali, Zhu[2], Ran Li[6], Jie Xu[1], Leshi, Chen[7], Yongliang Zhu[8], Long Zhang [5]*, Zhenjiang Bai[4]*, Fangfang Zhou[1 2 3]*

[1] Center of Clinical Laboratory, the First Affiliated Hospital of Soochow University, Suzhou, China.

[2] The institutes of Biology and Medical Science, Suzhou Medical College, Soochow University, Suzhou, China.

[3] Biomedical Basic Research Center (BBRC) of Jiangsu Province, Suzhou, China.

[4] Pediatric Intensive Care Unit, Children's Hospital of Soochow University, Suzhou, China.

[5] Life Sciences Institute and State Key Laboratory of Transvascular Implantation Devices of the Second Affiliated Hospital of Zhejiang University School of Medicine, Zhejiang University, Hangzhou, China.

[6] School of Medicine, Hangzhou City University, Hangzhou, China.

[7] State Key Laboratory of Genetic Engineering, School of Life Sciences, Fudan University, Shanghai, China

[8] Laboratory of Gastroenterology Department, Second Affiliated Hospital of Zhejiang University School of Medicine, Hangzhou, China.

# Equal contribution

* Corresponding author

Address correspondence to:

Correspondence should be addressed to F.Z. (zhoufangfang@suda.edu.cn), Z.B.(18913510429@163.com), L.Z. (L_Zhang@zju.edu.cn)

Supplementary Materials and Methods

RNA Extraction, Library Preparation, and Sequencing

Total RNA was extracted from fresh mouse lung tissues using Trizol reagent (Invitrogen) following the manufacturer's instructions. RNA integrity was examined by RNase-free agarose gel electrophoresis and evaluated with an Agilent 2100 Bioanalyzer. Eukaryotic mRNA was enriched using Oligo(dT) beads and reverse-transcribed into cDNA with the NEBNext Ultra RNA Library Prep Kit for Illumina. After end repair, A-tailing, and adapter ligation, cDNA fragments were purified with AMPure XP Beads and amplified by PCR. The final library was sequenced on an Illumina Novaseq 6000 platform by Gene Denovo Biotechnology Co., Ltd.

Proteomics sample preparation

Mouse lung tissues and human bronchial organoids were washed twice with PBS and lysed in buffer containing 8 M urea and 100 mM Tris (pH 8.5) supplemented with protease and phosphatase inhibitors (Roche, Switzerland). Protein concentration was determined using a BCA assay. After precipitation with cold acetone, proteins were dissolved in 50 mM ammonium bicarbonate, reduced with 5 mM TCEP (Sigma, USA), and alkylated with 10 mM iodoacetamide (Sigma, USA) in the dark. Trypsin digestion was performed at 37 °C for 16 h at an enzyme-to-protein ratio of 1:50. Digestion was terminated with formic acid. Peptides were desalted using SPE tubes (8B-S100-AAK, Phenomenex, USA), dried, and fractionated with Hp-RP StageTips.

HPLC-MS/MS analysis and database searching

Mass spectrometry was performed on an Orbitrap Fusion Lumos Tribrid mass spectrometer coupled with an Easy-nLC 1200 system. Mobile phases consisted of water and 80% acetonitrile, both containing 0.1% formic acid. Peptides were separated with a linear gradient. MS data were acquired in data-dependent mode. Raw files were processed using MaxQuant (v 2.0.3.0) and searched against the UniProtKB mouse or human database. Trypsin/P was set as the cleavage enzyme with up to two missed cleavages allowed. Carbamidomethylation was set as a fixed modification, and oxidation and protein N-terminal acetylation were variable modifications.

Transcriptome data processing

Raw reads were filtered using fastp (v 0.18.0) to remove low-quality reads and adapters. Clean reads were mapped to the rRNA database using Bowtie2 (v 2.2.8), and rRNA reads were removed. Remaining reads were aligned to the reference genome using HISAT2 (v 2.4). Gene expression levels were calculated as FPKM and TPM. Transcripts were assembled using StringTie (v 1.3.1).

Bioinformatics analysis

Samples with more than 50% missing values were excluded. Heatmaps were generated using the pheatmap package in R. PCA and Venn diagrams were constructed using the BioLadder online tool. Differential expression analysis was performed using the limma package. Genes with adjusted p-value < 0.05 and $|logFC| > 1$ were defined as differentially expressed genes. GO and KEGG enrichment analyses were conducted using clusterProfiler and DAVID. Immune infiltration was analyzed using CIBERSORT with the LM22 signature matrix. GSEA was performed using the gseKEGG function. Time-series clustering was performed using the Mfuzz package. Functional enrichment and interaction networks were analyzed using Metascape, Circos, and Cytoscape.

XGBoost model construction

The GEO dataset GSE246622 was downloaded using the GEOquery package. Samples were randomly divided into training and test sets at a 7:3 ratio. The XGBoost model was constructed using the XGBoost R package with the objective set to “binary:logistic” and max_depth = 6. Model performance was evaluated based on AUC value.

Real-time quantitative PCR (qPCR)

Total RNA was prepared using FreeZol Reagent (Vazyme, R711-02). A total of 500 ng of RNA was reverse-transcribed using TransScript® Uni All-in-One First-Strand cDNA Synthesis SuperMix for qPCR (One-Step gDNA Removal) (TransGen, AU341-02). Real-time PCR was conducted using PerfectStart® Green qPCR SuperMix (TransGen, AQ601-01-V2) with a StepOne Plus real-time PCR system. Relative expression of all murine target genes was determined by normalization to the internal reference gene *Gadph* rRNA. The following qPCR primers were used:

Gadph forward: 5’-GGCCTTCCGTGTTCCTACC-3’;

Gadph reverse: 5’-AGCCCAAGATGCCCTTCAGT-3’;

Cxcl10 forward: 5’-GTCCTAATTGCCCTTGGTCTTCT-3’;

Cxcl10 reverse: 5’-TCGCACCTCCACATAGCTTACAG-3’;

Cxcl9 forward: 5’-CGAGGCACGATCCACTACAA-3’;

Cxcl9 reverse: 5’-GAGTCCGGATCTAGGCAGGT-3’;

Il6 forward: 5’-ACCACTTCACAAGTCGGAGG-3’;

Il6 reverse: 5’-CAGAATTGCCATTGCACAAC-3’;

Il1b forward: 5’-CAACTGGTACATCAGCACCTCAC-3’;

Il1b reverse: 5’-ATTAGAAACAGTCCAGCCCATAC-3’;

Tnf forward: 5’-CAGCCTCTTCTCATTCCTGC-3’;

Tnf reverse: 5’-AGGGTCTGGGCCATAGAACT-3’;

Saa1 forward: 5’-GATCACCAGATCTGCCCAGG-3’;

Saa1 reverse: 5’-GCAGGGAGCAGAAGACCAG-3’;

Orm1 forward: 5’-CGAGTACAGGCAGGCAATTCA-3’;

Orm1 reverse: 5’-ACCTATTGTTTGAGACTCCCGA-3’;

Gpx1 forward: 5’-AGTCCACCGTGTATGCCTTCT-3’;

Gpx1 reverse: 5’-GAGACGCGACATTCTCAATGA-3’;

Nos2 forward: 5’-GTTCTCAGCCCAACAATACAAGA-3’;

Nos2 reverse: 5’-GTGGACGGGTCGATGTCAC-3’;

Vcam1 forward: 5’- AGTTGGGGATTCGGTTGTTCT -3’;

Vcam1 reverse: 5’-CCCCTCATTCCTTACCACCC-3’;

Icam1 forward: 5’-GTGATGCTCAGGTATCCATCCA-3’;

Icam1 reverse: 5’-CACAGTTCTCAAAGCACAGCG-3’;

Itgb3 forward: 5’-GGCGTTGTTGTTGGAGAGTC-3’;

Itgb3 reverse: 5’-CTTCAGGTTACATCGGGGTGA-3’;

Itga2b forward: 5’-TCCGTCTATGCAGGTCCCAAT-3’;

Itga2b reverse: 5’-CACGCTTCCATGTTTGTCCTT-3’;

Fn1 forward: 5’-ATGTGGACCCCTCCTGATAGT-3’;

Fn1 reverse: 5’-GCCCAGTGATTTCAGCAAAGG-3’;

Cdc42 forward: 5’-CCCATCGGAATATGTACCAACTG-3’;

Cdc42 reverse: 5'-CGGTCGTAGTCTGTCATAATCCT-3';

Itgb5 forward: 5'-GAAGTGCCACCTCGTGTGAA-3';

Itgb5 reverse: 5'-GGACCGTGGATTGCCAAAGT-3';

Itgam forward: 5'-TACTTCGGGCAGTCTCTGAGTG-3';

Itgam reverse: 5'-ATGGTTGCCTCCAGTCTCAGCA-3';

RSV forward: 5'- GAATTGCAGTTGCTCATGCAA-3';

RSV reverse: 5'- TGGCGATTGCAGATCCAACA -3'.

Immunoblot analysis

Cells were lysed on ice for 10 min using 1 mL of lysis buffer (20 mM Tris-HCl, pH 7.4, 2 mM EDTA, 25 mM NaF, 1% Triton X-100) supplemented with protease inhibitor cocktail (Sigma-Aldrich). Following centrifugation at 12×10^3 g for 15 min at 4°C, the protein concentration of the supernatant was determined. Equal amounts of protein were then separated by SDS-PAGE and transferred to a polyvinylidene difluoride (PVDF) membrane. Immunoblotting was performed using specific primary antibodies, followed by horseradish peroxidase (HRP)-conjugated secondary antibodies (anti-mouse or anti-rabbit, as appropriate). Protein bands were detected using enhanced chemiluminescence (ECL).

Antibodies used for immunoblotting were as follows: anti-FAK (Starter, S0B0831, 1:2000), anti-Phospho-FAK (Tyr397) (Starter, S0B6116, 1:2000), anti-Src (Starter, S0B1301, 1:2000), anti-Phospho-Src Family (Tyr416) (Starter, S0B1068, 1:2000), anti-AKT (Starter, S0B0114, 1:2000), anti- Phospho-Akt (Ser473) (Starter, S0B0363, 1:2000), anti- GSK3β (Starter, S0B1055, 1:2000), anti- Phospho-GSK-3β (Ser9) (Starter, S0B0518, 1:2000), anti-GAPDH (Abclonal, AC002, 1: 10,000), HRP-conjugated secondary antibodies (7076 (anti-mouse IgG) or 7074 (anti-rabbit IgG), Cell Signaling, 1:10,000).

Lung histopathology

Lung tissues were isolated from control or RSV-infected mice and immediately immersed in 4% paraformaldehyde (Beyotime, P0099) for 24 h fixation at 4°C. Following fixation, tissues were dehydrated through a graded ethanol series, cleared in xylene, and embedded in paraffin. Sections of 5 μm thickness were cut using a rotary microtome and mounted on positively charged glass slides.

After deparaffinization in xylene and rehydration through a descending ethanol series to distilled water, the sections were stained with Hematoxylin Staining Solution (Beyotime, C0107) for 5 min. Subsequently, they were rinsed under tap water, differentiated in 1% acid alcohol, and blued in 0.2% ammonia water. Counterstaining was performed with Eosin Staining Solution (Beyotime, C0109) for 30 s. Finally, the sections were dehydrated through an ascending ethanol series, cleared in xylene, and mounted with Neutral Balsam (Beyotime, C0173). Histopathological changes, including inflammatory cell infiltration, alveolar wall thickening, hemorrhage, and edema, were examined under a light microscope (Mshot).

Immunohistochemical Staining

For immunohistochemistry (IHC), paraffin-embedded lung tissue sections (5 μm thick) were deparaffinized in xylene and rehydrated through a graded ethanol series to distilled water. Antigen retrieval was performed by heat induction in boiling 10 mM sodium citrate buffer (pH 6.0) using a microwave oven for 20 min, followed by cooling at room temperature for 30 min. Endogenous peroxidase activity was blocked by incubating sections in 3% hydrogen peroxide (H_2O_2) in methanol for 15 min. Nonspecific binding sites were then blocked with 3% bovine serum albumin (BSA; Sigma, A7906) in PBS for 1 h at room temperature in a humidified chamber. Sections were subsequently incubated overnight at 4 °C with primary antibodies diluted in 3% BSA/PBS, including anti-Fibronectin (*Fn1*; Starter, S-1475-79) and anti-CD11b (*Itgam*; Starter, S0B2073P), both used at 1:200 dilution. After three 5 min washes with PBS, sections were incubated for 1 h at room temperature with appropriate horseradish peroxidase (HRP)-conjugated secondary antibodies diluted 1:200 in 3% BSA/PBS. Following three additional PBS washes, immunoreactivity was visualized using a DAB Horseradish Peroxidase Color Development Kit (Beyotime, P0203) according to the manufacturer's instructions.

Flow Cytometry

Single-cell suspensions were prepared from mouse lung tissues harvested at the indicated time points. Tissue dissociation was performed mechanically, followed by enzymatic digestion using collagenase I (5 mg/mL, Biosharp, BS163) and DNase I (1 mg/mL, BBI, B100649). Lymphocytes were then isolated from the single-cell suspension by centrifugation over a discontinuous Percoll

density gradient (40% and 80%), and the lymphocyte layer at the interface was collected. For intracellular cytokine detection, the isolated lymphocytes were first stimulated in vitro with 1× Cell Stimulation Cocktail (Invitrogen, 00-4970-93) for 4-6 h, followed by surface staining, fixation, and permeabilization for intracellular staining. The following fluorochrome-conjugated antibodies were used at a 1:200 dilution: APC anti-CD45 (Biolegend, #368512), FITC anti-CD3 (Biolegend, #100203), and PE anti-CD4 (Biolegend, #1004407) for surface markers, and APC/Cyanine7 anti-IFN-γ (Biolegend, #505850) and PE/Cyanine7 anti-IL-4 (Biolegend, #504118) for intracellular staining. Data were acquired on a BD FACSCanto II flow cytometer and analyzed using FlowJo software (version 10.8.1). Upon stimulation, Th1 cells were defined as $CD3^{+}CD4^{+}IFN\text{-}\gamma^{+}$ and Th2 cells as $CD3^{+}CD4^{+}IL\text{-}4^{+}$.

Supplementary-Figure legend

Fig S1. Fuzzy c-Means Clustering Reveals Temporal Gene Expression Patterns in RSV-Induced Lung Injury

At each time point, the quantitative mRNA data from multiple mice were classified into 6 different expression clusters using the fuzzy c-means clustering method, in order to demonstrate the relative transcriptional expression changes in the mouse model infected with RSV. The pathways corresponding to this cluster of genes obtained through KEGG enrichment analysis are on the right side.

Fig S2. Fuzzy c-Means Clustering Reveals Temporal Protein Expression Patterns in RSV-Induced Lung Injury

Fuzzy c-means clustering of quantified proteomic data from multiple mice per time point (0, 3, 7, 14 dpi) identified 6 expression clusters, showing relative translational expression alterations in RSV-infected mice. Corresponding pathways from KEGG enrichment analysis are on the right.

Fig S3. Fuzzy c-Means Clustering of Protein Expression in RSV-Infected Human Bronchial Organoids

By performing fuzzy c-means clustering on the quantitative proteomic data obtained from infected human lung bronchial organoids at each time point (0, 3, 7, and 14 days after RSV infection), six

expression clusters were identified, revealing the relative translation expression changes in RSV-infected human lung bronchial organoids. The relevant pathways obtained from KEGG enrichment analysis are shown on the right side.

Fig. S4. Functional and network analysis of lung injury-associated multi-omics signatures.

(A) Enrichment network of lung injury-related features across multi-omics datasets. Circular nodes represent significantly enriched biological processes (color-coded by functional category). Elliptical nodes denote signaling pathways (distinct colors indicate different pathways). Analyzed clusters include: transcriptomic clusters 3 and 4 (Lung-Transcriptome), proteomic clusters 1 and 6 (Lung-Proteome), and proteomic clusters 5 and 6 (Organoid-Proteome) - totaling six gene clusters. (Generated using Metascape).

(B) Protein-protein interaction (PPI) network of hub genes driving lung injury pathogenesis. Node color intensity correlates with degree centrality (darker hues means higher connectivity), reflecting key topological roles in the network.

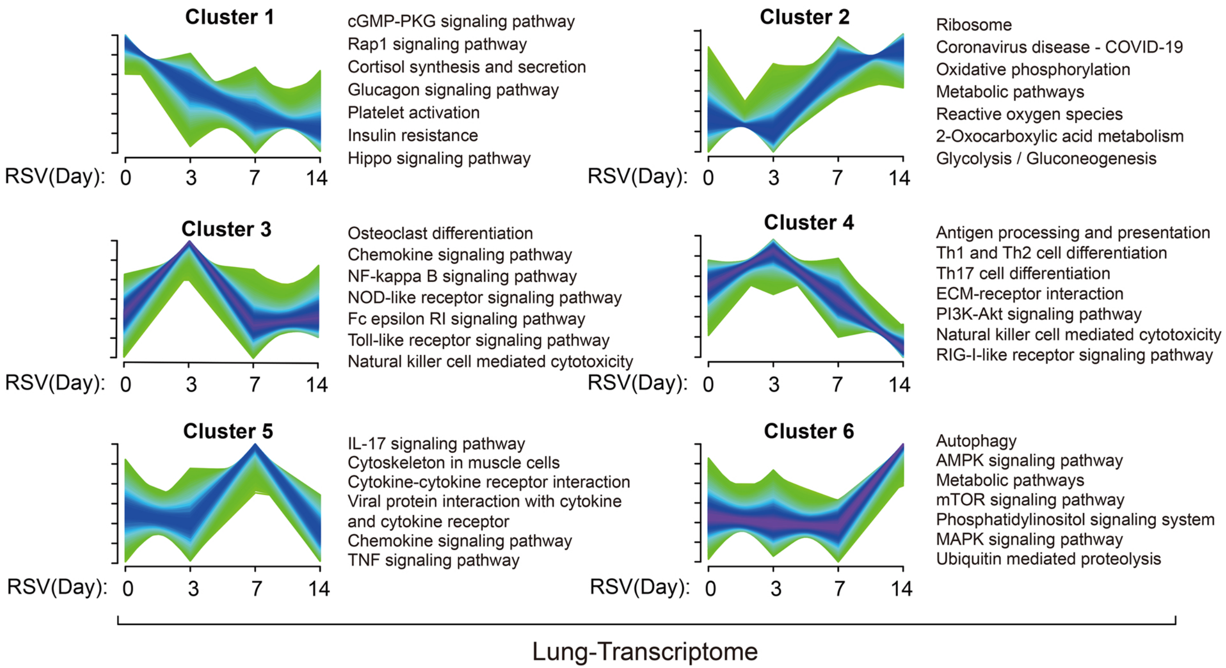
Cluster 1
cGMP-PKG signaling pathway
Rap1 signaling pathway
Cortisol synthesis and secretion
Glucagon signaling pathway
Platelet activation
Insulin resistance
Hippo signaling pathway
RSV(Day): 0 3 7 14
Cluster 2
Ribosome
Coronavirus disease - COVID-19
Oxidative phosphorylation
Metabolic pathways
Reactive oxygen species
2-Oxocarboxylic acid metabolism
Glycolysis / Gluconeogenesis
RSV(Day): 0 3 7 14
Cluster 3
Osteoclast differentiation
Chemokine signaling pathway
NF-kappa B signaling pathway
NOD-like receptor signaling pathway
Fc epsilon RI signaling pathway
Toll-like receptor signaling pathway
Natural killer cell mediated cytotoxicity
RSV(Day): 0 3 7 14
Cluster 4
Antigen processing and presentation
Th1 and Th2 cell differentiation
Th17 cell differentiation
ECM-receptor interaction
PI3K-Akt signaling pathway
Natural killer cell mediated cytotoxicity
RIG-I-like receptor signaling pathway
RSV(Day): 0 3 7 14
Cluster 5
IL-17 signaling pathway
Cytoskeleton in muscle cells
Cytokine-cytokine receptor interaction
Viral protein interaction with cytokine and cytokine receptor
Chemokine signaling pathway
TNF signaling pathway
RSV(Day): 0 3 7 14
Cluster 6
Autophagy
AMPK signaling pathway
Metabolic pathways
mTOR signaling pathway
Phosphatidylinositol signaling system
MAPK signaling pathway
Ubiquitin mediated proteolysis
RSV(Day): 0 3 7 14
Lung-Transcriptome

Figure S1

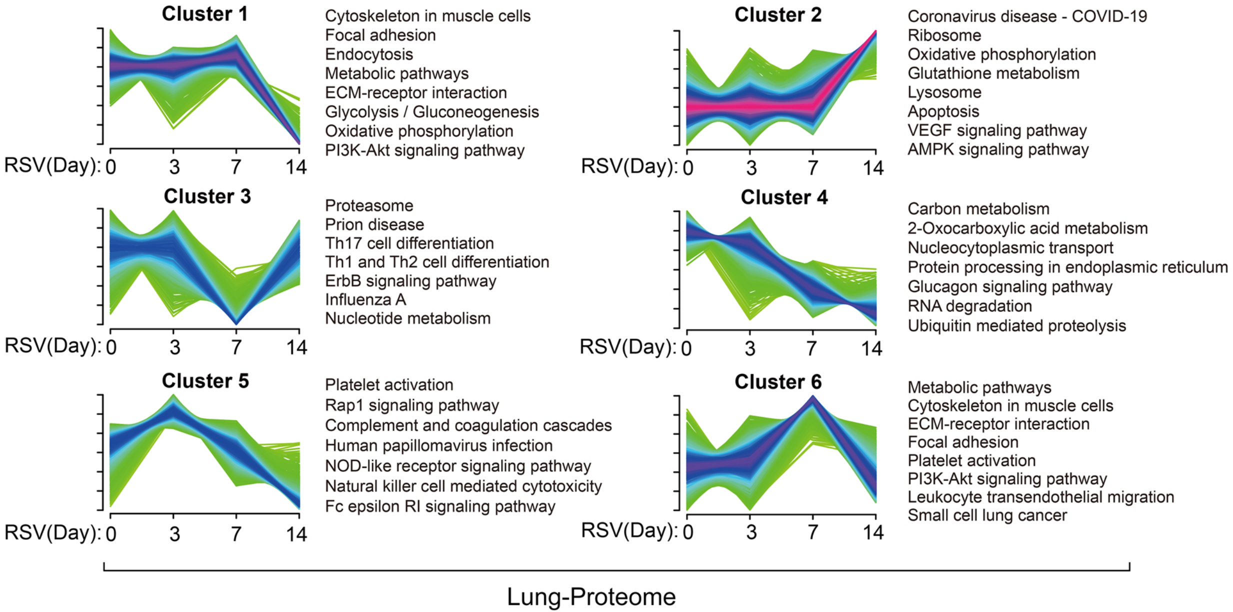
Cluster 1
Cytoskeleton in muscle cells
Focal adhesion
Endocytosis
Metabolic pathways
ECM-receptor interaction
Glycolysis / Gluconeogenesis
Oxidative phosphorylation
PI3K-Akt signaling pathway
RSV(Day): 0 3 7 14
Cluster 2
Coronavirus disease - COVID-19
Ribosome
Oxidative phosphorylation
Glutathione metabolism
Lysosome
Apoptosis
VEGF signaling pathway
AMPK signaling pathway
RSV(Day): 0 3 7 14
Cluster 3
Proteasome
Prion disease
Th17 cell differentiation
Th1 and Th2 cell differentiation
ErbB signaling pathway
Influenza A
Nucleotide metabolism
RSV(Day): 0 3 7 14
Cluster 4
Carbon metabolism
2-Oxocarboxylic acid metabolism
Nucleocytoplasmic transport
Protein processing in endoplasmic reticulum
Glucagon signaling pathway
RNA degradation
Ubiquitin mediated proteolysis
RSV(Day): 0 3 7 14
Cluster 5
Platelet activation
Rap1 signaling pathway
Complement and coagulation cascades
Human papillomavirus infection
NOD-like receptor signaling pathway
Natural killer cell mediated cytotoxicity
Fc epsilon RI signaling pathway
RSV(Day): 0 3 7 14
Cluster 6
Metabolic pathways
Cytoskeleton in muscle cells
ECM-receptor interaction
Focal adhesion
Platelet activation
PI3K-Akt signaling pathway
Leukocyte transendothelial migration
Small cell lung cancer
RSV(Day): 0 3 7 14
Lung-Proteome

Figure S2

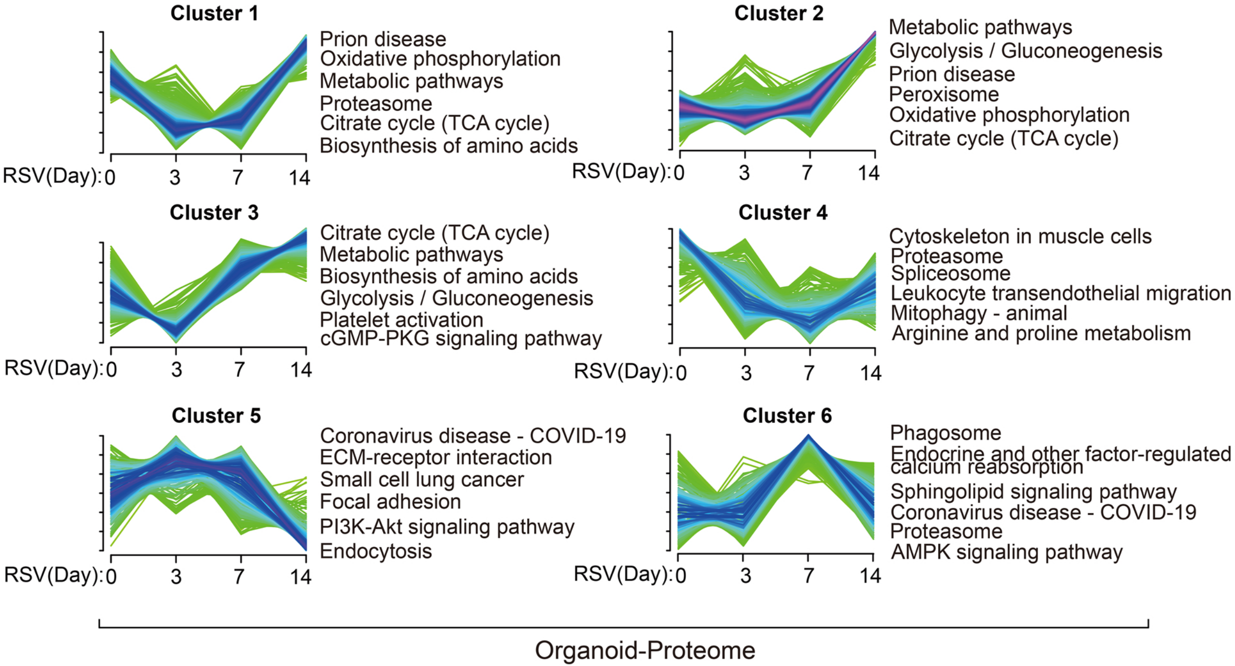
Cluster 1
Prion disease
Oxidative phosphorylation
Metabolic pathways
Proteasome
Citrate cycle (TCA cycle)
Biosynthesis of amino acids
RSV(Day): 0 3 7 14
Cluster 2
Metabolic pathways
Glycolysis / Gluconeogenesis
Prion disease
Peroxisome
Oxidative phosphorylation
Citrate cycle (TCA cycle)
RSV(Day): 0 3 7 14
Cluster 3
Citrate cycle (TCA cycle)
Metabolic pathways
Biosynthesis of amino acids
Glycolysis / Gluconeogenesis
Platelet activation
cGMP-PKG signaling pathway
RSV(Day): 0 3 7 14
Cluster 4
Cytoskeleton in muscle cells
Proteasome
Spliceosome
Leukocyte transendothelial migration
Mitophagy - animal
Arginine and proline metabolism
RSV(Day): 0 3 7 14
Cluster 5
Coronavirus disease - COVID-19
ECM-receptor interaction
Small cell lung cancer
Focal adhesion
PI3K-Akt signaling pathway
Endocytosis
RSV(Day): 0 3 7 14
Cluster 6
Phagosome
Endocrine and other factor-regulated calcium reabsorption
Sphingolipid signaling pathway
Coronavirus disease - COVID-19
Proteasome
AMPK signaling pathway
RSV(Day): 0 3 7 14
Organoid-Proteome

Figure S3

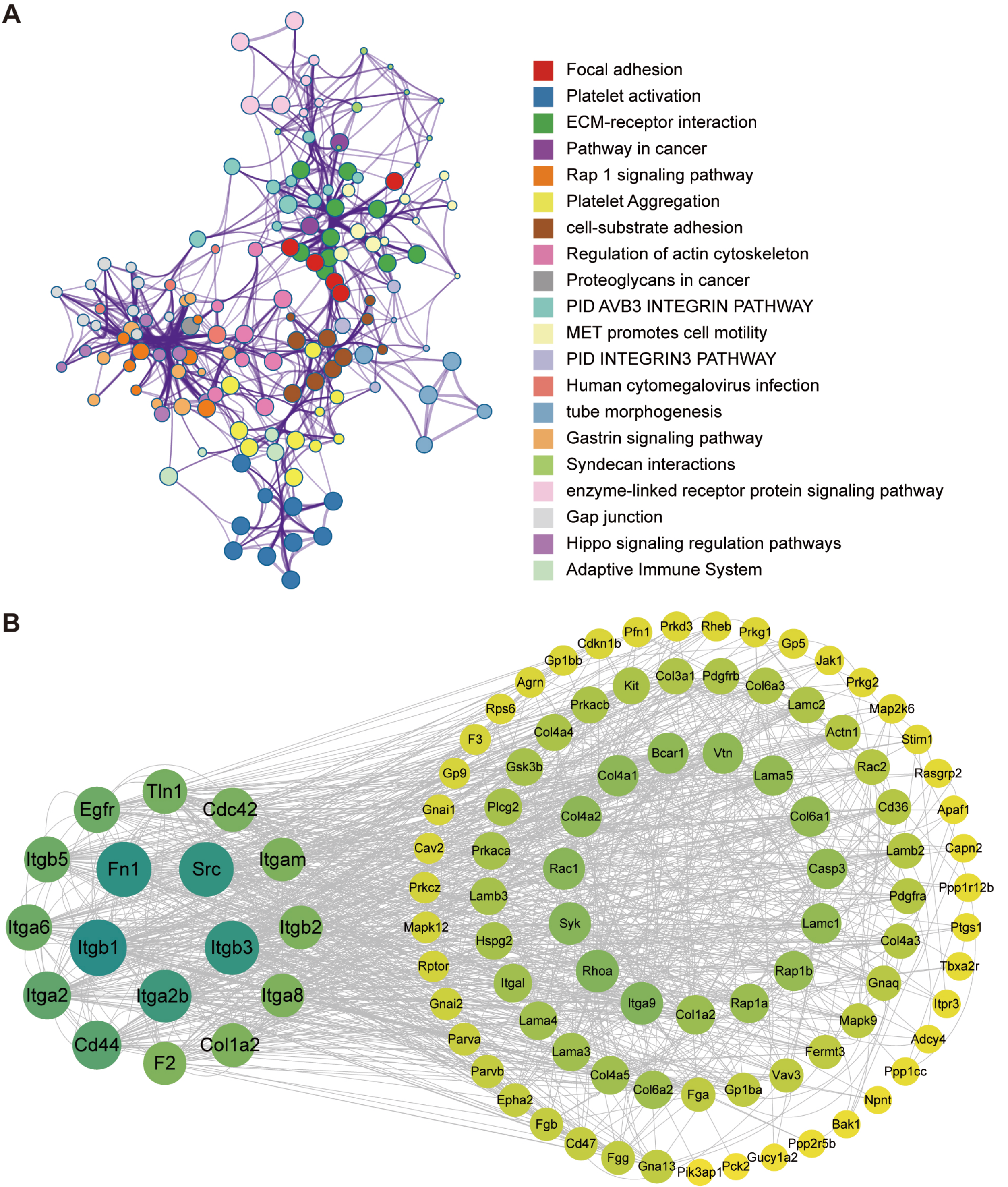
A
Focal adhesion
Platelet activation
ECM-receptor interaction
Pathway in cancer
Rap 1 signaling pathway
Platelet Aggregation
cell-substrate adhesion
Regulation of actin cytoskeleton
Proteoglycans in cancer
PID AVB3 INTEGRIN PATHWAY
MET promotes cell motility
PID INTEGRIN3 PATHWAY
Human cytomegalovirus infection
tube morphogenesis
Gastrin signaling pathway
Syndecan interactions
enzyme-linked receptor protein signaling pathway
Gap junction
Hippo signaling regulation pathways
Adaptive Immune System
B
Tln1
Egfr
Cdc42
Itgb5
Fn1
Src
Itgam
Itga6
Itgb1
Itgb3
Itgb2
Itga2
Itga2b
Itga8
Cd44
F2
Col1a2
Prkd3
Rheb
Pfn1
Prkg1
Cdkn1b
Gp5
Gp1bb
Jak1
Col3a1
Pdgfrb
Kit
Col6a3
Agrn
Prkg2
Prkacb
Lamc2
Rps6
Map2k6
Col4a4
Actn1
F3
Stim1
Bcar1
Vtn
Gsk3b
Rac2
Gp9
Col4a1
Lama5
Rasgrp2
Plcg2
Cd36
Gnai1
Apaf1
Col4a2
Col6a1
Cav2
Prkaca
Lamb2
Capn2
Rac1
Casp3
Prkcz
Lamb3
Pdgfra
Ppp1r12b
Syk
Lamc1
Mapk12
Ptgs1
Hspg2
Col4a3
Rptor
Rhoa
Tbxa2r
Rap1b
Itgal
Gnaq
Itga9
Rap1a
Itpr3
Gnai2
Col1a2
Lama4
Mapk9
Parva
Adcy4
Lama3
Fermt3
Parvb
Ppp1cc
Col4a5
Vav3
Col6a2
Gp1ba
Fga
Epha2
Npnt
Fgb
Bak1
Ppp2r5b
Cd47
Fgg
Gucy1a2
Pck2
Gna13
Pik3ap1

Figure S4
